# Supplementary material for: Redefined ion association constants have consequences for calcium phosphate nucleation and biomineralization
Source: Nat Commun. 2024 Apr 18;15:3359. doi: 10.1038/s41467-024-47721-7 (PMC11026415; doi:10.1038/s41467-024-47721-7)
Supplement: Supplementary file 1 — Supplementary Information File [file 41467_2024_47721_MOESM1_ESM.pdf]

Supplementary Information

Redefined ion association constants have consequences for calcium phosphate nucleation and biomineralization

David P. McDonogh,[1] Julian D. Gale,[2] Paolo Raiteri,[2,*] and Denis Gebauer[1,*]

1. Institute of Inorganic Chemistry, Leibniz University Hannover, Callinstr. 9, 30167 Hannover, Germany, gebauer@acc.uni-hannover.de
2. Curtin Institute for Computation and School of Molecular and Life Sciences, Curtin University, P.O. Box U1987, Perth, WA 6845, Australia, p.raiteri@curtin.edu.au

Contents

1. Acidified vs. non-acidified titrations 2
2. Binding evaluation results 2
3. AMOEBA Force field parameters 4
4. Titrations at 37°C 8
5. Supplementary Figures 9
6. Supplementary Tables 34
7. Supplementary References 37

1. Acidified vs. non-acidified titrations

As mentioned in the main text, the calcium solution titrated into the phosphate buffer was acidified as it was seen that premature phase separation occurs in the non-acidified system. This is demonstrated in Figures 1 and Supplementary Figure 1; instead of a linear pre-nucleation slope, a sharp peak at nucleation and subsequent stabilisation at a free calcium concentration determined by the nucleated phase, the non-acidified titrations have a bent pre-nucleation regime which curves directly into the solubility. Such direct titration indicates that phase separation has already occurred before the entire reaction volume is at the critical supersaturation.

The curved pre-nucleation regime in the non-acidified titrations binds more calcium than in the linear acidified case. This calcium is already in the separated phase. As seen in the light microscopy images (Supplementary Figure 2), this produces a whitish translucent gel in the calcium dosing tip. The precipitated phase is difficult to detect visually and so may have been missed by previous studies. As calcium ions are removed from solution by this phase, ion association is overestimated if it is not recognized that Ca^{2+} is being sequestered from the solution.

2. Binding evaluation results

As mentioned in the main text, the value for K_1 was calculated directly from the data at pH 4.3 as the influence of other phosphate species on the binding is minimal at this pH. This was done for each experiment through the law of mass action and then averaging the region of each curve where K_1 had reached a constant value. K_1 was determined to be 4.5 ± 2.4. The complete set of ion association constants is summarised in Table 1. Three sets of binding constants are evaluated and the results overlayed onto the experimental data as shown in Supplementary Figures 9 to 11, the best fit, and two limits based on the errors in the best fit where the calculated curves begin to deviate – the true values lie within this range. The "Limit 1" and "Limit 2" cases use a combination of the minimum and maximum K_2 and K_3 values and *vice versa*; the first pairing constant was taken as 4.5 as changing this has only a small effect on the results. The best fit and errors were determined by manually testing combinations of binding constants and visually finding ion association triplets where the curves begin to deviate (see Supplementary Movies). The errors on the ion association constants shown in Table 1 were then taken to be the largest deviation for K_2 and K_3, respectively. A global, mathematical minimisation of the deviation is hindered by a multitude of local minima, thus not rendering it any more rigorous, in a practical sense. In fact, we expect any mathematical minimisation of the deviation to result in smaller errors than those estimated by us. In any case, the error estimation from the global fit dominates, as the multiple measurements at the individual pH values show very good agreement, with the relative error generally well under 5%. The approach used from evaluating the limit cases thus provides a generous margin, easily compensating for any experimental uncertainty, and is eventually more conservative than any mathematical minimisation. Considering how the ion pairing constants compare to the literature values (Supplementary Table 1, see also discussion in main text and following here), even an error of 20% (or 50%) would not affect the main conclusions of this work. This is especially true for K_3, where we show a correction of two orders of magnitude to the literature value.

Several features can be observed in the Supplementary Figures 9, 10 and 11. For both models, the free calcium activity is overestimated for high pH values using the "Best Fit" and "Limit 2" constants

while the same is true for midrange pH values with “Best Fit” and “Limit 1” constants. Additionally, pH 7.8 and 7.3 show a curving behaviour, the calculated values skew away from the data once the added calcium concentration is ~0.3 M before swinging back in the direction of the titration data. Meanwhile the calculated binding profiles are linear at the other pH values. In all cases the Predictive Calculation lies closer to the experimental data. Notably the predicted cures are linear at all pH values. This occurs since this model does not rely on experimental concentrations for the calculation while the Direct Calculation does- curvature in the experimental data is then reflected in the calculation. Due to the dependence on the experimental data, the direct calculation is also more sensitive to changes in the ion association constants and displays greater errors.

The match between binding from literature sources and titrations can be directly compared. Using the values from Chughtai *et al.*, the binding is overestimated at all pH values (Supplementary Figure 12).[1] Using the Direct Calculation, the free calcium activity goes into the negative numbers at and above pH 8.8. While this is not chemically feasible, the mathematical setup of the model produces this result (discussed below). Changes to the calculated activity of calcium are smaller for the Predictive Calculation. However, using the Chughtai *et al.* values, the binding is still overestimated at all pH values as the predicted curves lie underneath the experimental ones. The overestimation at the lower pH indicates that the K_1 and K_2 values are also too large.

In Supplementary Figure 12, the Direct Calculation can result in unphysical negative activities since the approach used relies directly on the measured amounts of species in solution. It does not start from the added amounts and then subtract what the binding will remove. Rather the K_i values which match the measured data using given concentrations are found. When too large binding constants are used, the amount of bound species given for the given solution composition is overestimated. Subtracting this from the amount of added calcium then returns negative numbers.

The Predictive Calculation overcomes the limitation of the above, the concentrations of the reactants are decreased as the products increase – leading to additions and subtractions to the respective terms in the law of mass action. The final composition is then calculated from the initial reagent concentrations by rearranging the law of mass action to find the magnitude of the additions and subtractions. This then guarantees that the calculated equilibrium concentrations/activities lie within the positive numbers, and a chemically meaningful range.

As discussed in the main text, K_1 and K_2 fall within the range of literature values, but K_3 falls two orders of magnitude below the commonly used value. K_1 and K_2 can be measured at pH values where the respective phosphate species are dominant in solution leading to the plethora of reported ion association constants. Especially for K_2 the consistency between literature values reflects the ease of measurement due to HPO_4^{2-} being in excess in an accessible pH range combined with the decent affinity for calcium leading to measurable binding at reasonable concentrations. Increasing the pH to where PO_4^{3-} exists solely would not only require working with extremely dilute solutions but would lead to calcium association with hydroxide. This was neglected in this work as the low ion pairing constant means leads to insignificant calcium binding at the pH values investigated (the value for $K_{Ca^{2+}+OH^-}$ is ~20.)[2]

3. AMOEBA Force field parameters

Parameters for the Ca^{2+}-PO_4^{3-}/HPO_4^{2-} interactions in XML format for OpenMM. The additional Buckingham interactions used for this work can be added at the end of the file within the Script section using the OpenMM CustomNonbondedForce class. The addTabulatedFunction method can also be used to add the potential between selected species. The specifics for the use of these functionalities depend on whether the OpenMM python, C++ or fortran APIs are used, and are therefore left to the user.


```
<ForceField>
  <Info>
    <Author> Paolo Raiteri </Author>
    <DateGenerated> 11-01-2023 </DateGenerated>
    <Reference>
      H2O
      Laury, M. L.; Wang, L.-P.; Pande, V. S.; Head-Gordon, T.; Ponder, J. W.
      Revised parameters for the AMOEBA polarizable atomic multipole water model.
      J. Phys. Chem. B 2015, 119, 9423- 9437.
    </Reference>
  </Info>

  <AtomTypes>
<!-- H2O -->
    <Type name="1"  class="1"  element=" O" mass="15.999" /> <!-- "Water_O" -->
    <Type name="2"  class="2"  element=" H" mass=" 1.008" /> <!-- "Water_H" -->

<!-- Ca -->
    <Type name="3"  class="3"  element="Ca" mass="40.078" /> <!--
"Calcium_Ion_Ca+2" -->

<!-- PO4 -->
    <Type name="29" class="29" element=" P" mass="30.974" /> <!-- "Phosphate_P" -->
    <Type name="30" class="30" element=" O" mass="15.995" /> <!-- "Phosphate_O" -->

<!-- HPO4 -->
    <Type name="31" class="31" element=" P" mass="30.974" /> <!-- "Hphosphate_P" --
>
    <Type name="32" class="32" element=" O" mass="15.995" /> <!-- "Hphosphate_O" --
>
    <Type name="33" class="33" element=" O" mass="15.995" /> <!-- "Hphosphate_OH" -
->
    <Type name="34" class="34" element=" H" mass=" 1.008" /> <!-- "Hphosphate_H" --
>
  </AtomTypes>

  <Residues>
    <Residue name="HOH">
      <Atom name="O"  type="1" />
      <Atom name="H1" type="2" />
      <Atom name="H2" type="2" />
      <Bond atomName1="H1" atomName2="O" />
      <Bond atomName1="H2" atomName2="O" />
    </Residue>
    <Residue name="Ca">
      <Atom name="Ca" type="3"/>
    </Residue>
    <Residue name="PO4">
      <Atom name="P"  type="29" />
      <Atom name="O1" type="30" />
      <Atom name="O2" type="30" />
      <Atom name="O3" type="30" />
      <Atom name="O4" type="30" />
```

```
      <Bond atomName1="O1" atomName2="P" />
      <Bond atomName1="O2" atomName2="P" />
      <Bond atomName1="O3" atomName2="P" />
      <Bond atomName1="O4" atomName2="P" />
    </Residue>
    <Residue name="HPO4">
      <Atom name="P"  type="31" />
      <Atom name="O1" type="32" />
      <Atom name="O2" type="32" />
      <Atom name="O3" type="32" />
      <Atom name="O4" type="33" />
      <Atom name="H4" type="34" />
      <Bond atomName1="O1" atomName2="P" />
      <Bond atomName1="O2" atomName2="P" />
      <Bond atomName1="O3" atomName2="P" />
      <Bond atomName1="O4" atomName2="P" />
      <Bond atomName1="H4" atomName2="O4" />
    </Residue>
  </Residues>

<!-- Bond stretching parameters, units: nm and kj/mol/nm^2 -->
  <AmoebaBondForce bond-cubic="-25.5" bond-quartic="379.3125">
<!-- H2O -->
    <Bond class1="1"  class2="2"  length="0.09572" k="232986.04"/>

<!-- PO4 -->
    <Bond class1="29" class2="30" length="0.15660" k="146440.00"/>

<!-- HPO4 -->
    <Bond class1="31" class2="32" length="0.15330" k="146440.00"/>
    <Bond class1="31" class2="33" length="0.16920" k="146481.84"/>
  </AmoebaBondForce>

<!-- Angle bending parameters, units: kj/mol/deg^2 and deg -->
  <AmoebaAngleForce angle-cubic="-0.014" angle-quartic="5.6e-05"
                    angle-pentic="-7e-07" angle-sextic="2.2e-08">
<!-- H2O -->
    <Angle class1="2"  class2="1"  class3="2"  k="0.06206912" angle1="108.50"  />

<!-- PO4 -->
    <Angle class1="30" class2="29" class3="30" k="0.20392320" angle1="109.47"  />

<!-- HPO4 -->
    <Angle class1="32" class2="31" class3="32" k="0.19997219" angle1="115.00"  />
    <Angle class1="32" class2="31" class3="33" k="0.19997219" angle1="101.00"  />
    <Angle class1="31" class2="33" class3="34" k="0.04999942" angle1="111.00"  />
  </AmoebaAngleForce>

<!-- Out of plane bending parameters, units: kj/mol/deg^2 -->
  <AmoebaOutOfPlaneBendForce type="ALLINGER"
                             opbend-cubic="-0.014" opbend-quartic="5.6e-05"
                             opbend-pentic="-7e-07" opbend-sextic="2.2e-08">
  </AmoebaOutOfPlaneBendForce>

<!-- Torsion rotation parameters, units: kj/mol/deg -->
  <AmoebaTorsionForce torsionUnit="0.5">
<!-- HPO4 -->
    <Torsion class1="34" class2="33" class3="31" class4="32"
               amp1="  0.0000" angle1="  0.00000000"
               amp2="  0.0000" angle2="  3.14159265"
               amp3="  0.7029" angle3="  0.00000000" />
  </AmoebaTorsionForce>

<!-- Bond stretching-angle bending cross terms, units: kJ/mol/nm/deg -->
  <AmoebaStretchBendForce stretchBendUnit="1.0">
<!-- PO4 -->
    <StretchBend class1="30" class2="29" class3="30" k1="10.5155424" k2="
10.5155424" />
```

```
    <StretchBend class1="32" class2="31" class3="32" k1="10.5155424" k2="
10.5155424" />

<!-- HPO4 -->
    <StretchBend class1="32" class2="31" class3="33" k1="10.5155424" k2="
10.5155424" />
    <StretchBend class1="31" class2="33" class3="34" k1=" 9.4566857" k2="
9.4566857" />
  </AmoebaStretchBendForce>

<!-- van der Waals parameters, units: kJ/mol and nm (from kcal/mol and A) -->
  <AmoebaVdwForce type="BUFFERED-14-7" radiusrule="CUBIC-MEAN" radiustype="R-MIN"
                  radiussize="DIAMETER" epsilonrule="HHG"
                  vdw-13-scale="0.0" vdw-14-scale="1.0" vdw-15-scale="1.0" >
<!-- H2O -->
    <Vdw class="1"    sigma="0.34050"  epsilon="0.460240"   reduction="1.00" />
    <Vdw class="2"    sigma="0.26550"  epsilon="0.056484"   reduction="0.91" />

<!-- Ca -->
    <Vdw class="3"    sigma="0.35500"  epsilon="1.464400"   reduction="1.00" />

<!-- PO4 -->
    <Vdw class="29"   sigma="0.44500"  epsilon="1.631760"   reduction="1.00" />
    <Vdw class="30"   sigma="0.33600"  epsilon="0.468608"   reduction="1.00" />

<!-- HPO4 -->
    <Vdw class="31"   sigma="0.47500"  epsilon="1.631760"   reduction="1.00" />
    <Vdw class="32"   sigma="0.33600"  epsilon="0.468608"   reduction="1.00" />
    <Vdw class="33"   sigma="0.34050"  epsilon="0.468608"   reduction="1.00" />
    <Vdw class="34"   sigma="0.26550"  epsilon="0.056484"   reduction="0.91" />
  </AmoebaVdwForce>

  <AmoebaMultipoleForce  direct11Scale="0.0" direct12Scale="1.0"
direct13Scale="1.0"
                         direct14Scale="1.0" mpole12Scale="0.0"  mpole13Scale="0.0"
                         mpole14Scale="0.4"  mpole15Scale="0.8" mutual11Scale="1.0"
                         mutual12Scale="1.0" mutual13Scale="1.0"
mutual14Scale="1.0"
                         polar12Scale="0.0"  polar13Scale="0.0"  polar14Intra="0.5"
                         polar14Scale="1.0"  polar15Scale="1.0"  >
<!-- H2O -->
    <Multipole type="1"  kz="-2"  kx="-2"
              c0 ="-0.519660000"
              d1 =" 0.000000000" d2 =" 0.000000000" d3 =" 0.007556121"
              q11=" 0.000354031"
              q21=" 0.000000000" q22="-0.000390257"
              q31=" 0.000000000" q32=" 0.000000000" q33=" 0.000036226"  />
    <Multipole type="2"  kz="1"  kx="2"
              c0 =" 0.259830000"
              d1 ="-0.002042095" d2 =" 0.000000000" d3 ="-0.003078753"
              q11="-0.000034285"
              q21=" 0.000000000" q22="-0.000100241"
              q31="-0.000001895" q32=" 0.000000000" q33=" 0.000134526"  />

<!-- Ca -->
    <Multipole type="3"  kz="0"  kx="0"
              c0 =" 2.000000000"
              d1 =" 0.000000000" d2 =" 0.000000000" d3 =" 0.000000000"
              q11=" 0.000000000"
              q21=" 0.000000000" q22=" 0.000000000"
              q31=" 0.000000000" q32=" 0.000000000" q33=" 0.000000000"  />

<!-- PO4 -->
    <Multipole type="29" kz="30" kx="30"
              c0 =" 2.660760000"
              d1 =" 0.000000000" d2 =" 0.000000000" d3 =" 0.000000000"
              q11=" 0.000000000"
              q21=" 0.000000000" q22=" 0.000000000"
```

```
            q31=" 0.000000000" q32=" 0.000000000" q33=" 0.000000000"  />
    <Multipole type="30" kz="29" kx="30"
            c0 ="-1.415190000"
            d1 =" 0.000000000" d2 =" 0.000000000" d3 ="-0.010831728"
            q11="-0.000118611"
            q21=" 0.000000000" q22="-0.000118611"
            q31=" 0.000000000" q32=" 0.000000000" q33=" 0.000237221"  />

<!-- HPO4 -->
    <Multipole type="31" kz="32" kx="-32"
            c0 =" 2.607300000"
            d1 =" 0.000000000" d2 =" 0.000000000" d3 =" 0.000000000"
            q11=" 0.000000000"
            q21=" 0.000000000" q22=" 0.000000000"
            q31=" 0.000000000" q32=" 0.000000000" q33=" 0.000000000"  />
    <Multipole type="32" kz="31" kx="32"
            c0 ="-1.337360000"
            d1 =" 0.000000000" d2 =" 0.000000000" d3 =" 0.000000000"
            q11=" 0.000000000"
            q21=" 0.000000000" q22=" 0.000000000"
            q31=" 0.000000000" q32=" 0.000000000" q33=" 0.000000000"  />
    <Multipole type="33" kz="31" kx="34"
            c0 ="-0.813310000"
            d1 =" 0.016619339" d2 =" 0.000000000" d3 ="-0.009361674"
            q11=" 0.000541650"
            q21=" 0.000000000" q22="-0.000393916"
            q31="-0.000239191" q32=" 0.000000000" q33="-0.000147734"  />
    <Multipole type="34" kz="33" kx="31"
            c0 =" 0.218090000"
            d1 ="-0.000272526" d2 =" 0.000000000" d3 ="-0.004765770"
            q11="-0.000057910"
            q21=" 0.000000000" q22="-0.000077045"
            q31=" 0.000002016" q32=" 0.000000000" q33=" 0.000134955"  />

<!--############################ Polarisation ###########################-->
<!-- H2O -->
    <Polarize type="1"  polarizability="0.000837" thole="0.39" pgrp1="2" />
    <Polarize type="2"  polarizability="0.000496" thole="0.39" pgrp1="1" />

<!-- Ca -->
    <Polarize type="3"  polarizability="0.000550" thole="0.16" />

<!-- PO4 -->
    <Polarize type="29" polarizability="0.001828" thole="0.39" pgrp1="30" />
    <Polarize type="30" polarizability="0.000837" thole="0.39" pgrp1="29" />

<!-- HPO4 -->
    <Polarize type="31" polarizability="0.001828" thole="0.39" pgrp1="32"
pgrp2="33" />
    <Polarize type="32" polarizability="0.000837" thole="0.39" pgrp1="31" />
    <Polarize type="33" polarizability="0.000837" thole="0.39" pgrp1="31"
pgrp2="34" />
    <Polarize type="34" polarizability="0.000496" thole="0.39" pgrp1="33" />
  </AmoebaMultipoleForce>

<!-- Urey-Bradley term for water, units kj/mol/nm^2 -->
  <AmoebaUreyBradleyForce cubic="0.0" quartic="0.0"  >
<!-- H2O -->
    <UreyBradley class1="2" class2="1" class3="2" k="-3179.84" d="0.15326" />
  </AmoebaUreyBradleyForce>

  <Script>
  </Script>

</ForceField>
```

4. Titrations at 37°C

As mentioned in the main text, the titration into the plateaus seen at 37°C will likely lead to a slight overestimation of the binding constants – this should be most evident at pH 11.3 and affect the value of K_3. At pH 8.3 the two sets of data are in good agreement; here K_2 dominates the binding so this value should not change by a large amount. The tricky nature of the titrations, and CaP ion association, is well illustrated here where a change of less than 20°C requires careful consideration of the data. Various HCl (0.001-0.1 M) and NaOH (0.01-0.1) concentrations were used, however, no effect could be seen on the data – this mixed dataset was used for the evaluation. As no experiments were performed at low pH the value for K_1 cannot be calculated, however, based on values given in the literature it is assumed that it changes by a negligible amount.[1, 3, 4] This is justified as this binding equilibrium is the lowest by several orders of magnitude. Any knock-on effects on the calculated Gibbs free energy are disregarded for the same reasons. The ion association constants are summarised in the Supplementary Table 3 and the fits shown in Supplementary Figures 23-25. These fits are then used to calculate the thermodynamic parameters for the ion association. Overall the reaction is thermodynamically favourable, and is driven by the entropic contribution.

For the titrations, error propagation for the thermodynamic quantities was done using partial differentials of the equations and the errors on the ion association constants given in Tables 1 and Supplementary Table 3. The errors on the ΔG^0 values at 37°C are 2.8, 0.2 and 0.3 kJ/mol for K_1, K_2 and K_3, respectively. While the errors on the Gibbs Free Energy values are small, representing the excellent agreement between repeat measurements seen in in the titrations, the standard enthalpy and standard entropy values show much larger deviations due to the limited number of temperatures titrations were performed at. This again highlights the utility of coupling such measurements with simulation, which allows such ranges to be narrowed. The uncertainties for simulations are the 95% confidence intervals obtained from the fits.

5. Supplementary Figures

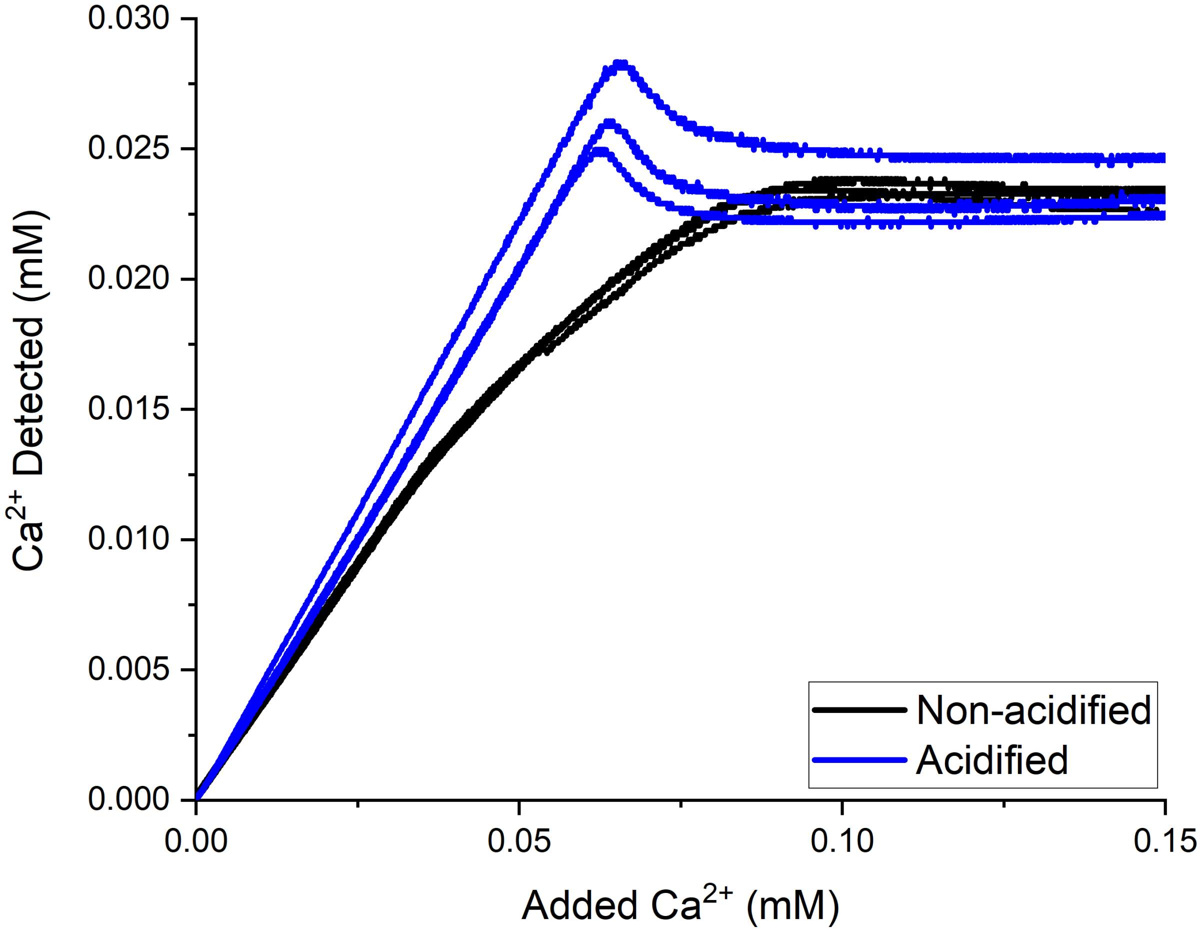

Supplementary Figure 1 Comparison of titrations with and without acidification of the added calcium solution at pH 9.8. The acidification prevents phase separation due to local overconcentration; less calcium is bound in the acidified conditions, and a maximum calcium concentration is seen at nucleation. Non-acidified titrations proceed directly into a solubility plateau – the free calcium concentration pre-nucleation is less as the phase at the calcium tip scavenges the ions as they are added. It can be seen that the premature phase separation leads to a distinctly increased, arguably non-equilibrated pre-nucleation calcium binding, thus overestimating the corresponding ion association constants.

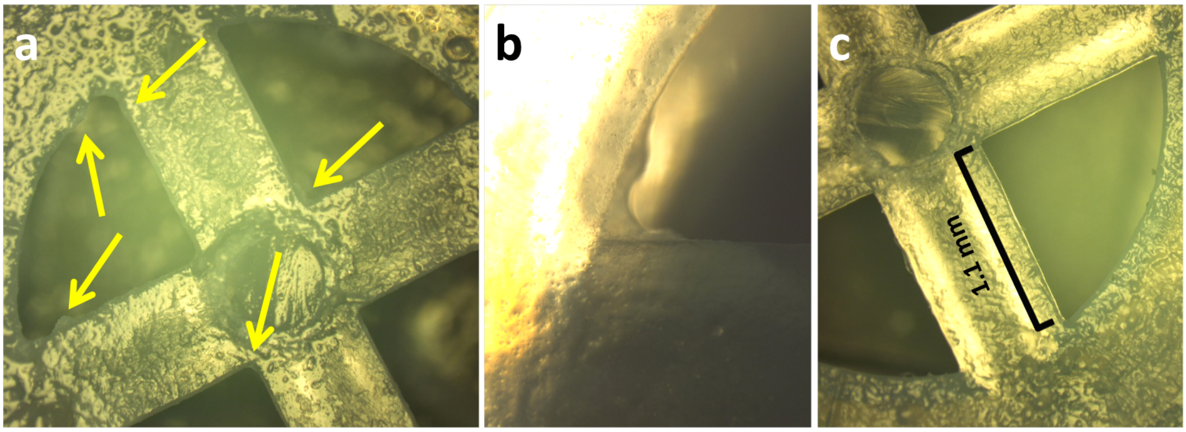

Supplementary Figure 2 Phase separated CaP from early stages of non-acidified titration. Calcium dosing tip from a titration at pH 9.8 without acidification of the added solution viewed in a Keyence VHX-600 Digital Microscope equipped with a VHZ100UR Zoom Lens. **(a)** The yellow arrows point to deposits of a calcium phosphate phase inside the dosing tip. Some larger deposits on the flat sections can be seen while most of the corners have material in them. **(b)** Close-up of a corner in the tip where a deposit can be seen. **(c)** The same dosing tip after treatment with 0.1 M HCl solution. The deposited calcium phosphate phase has been removed. The material seen here has dried during investigation; the material is hydrated and more translucent on dosing tips directly taken out of solution, making this extremely difficult to spot by eye.

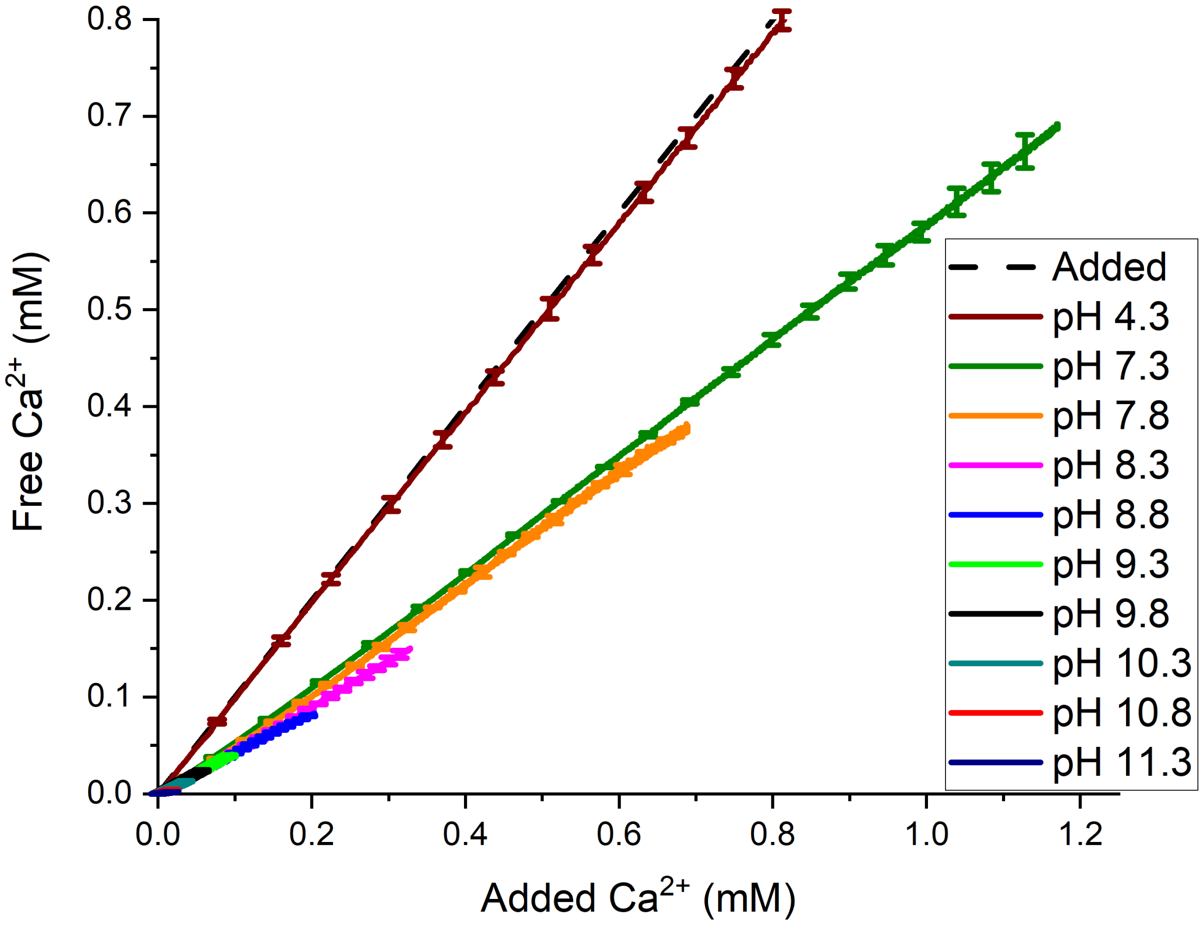

Supplementary Figure 3 Pre-nucleation regimes for Ca^{2+} to phosphate buffer addition at all measured pH values. Lines are averages of three experiments with error bars (±standard deviation, N=3) shown at 20 points until the end of the data or frame, curves extending above the y-axis limit may have less. As pH increases the pre-nucleation slope decreases and the pre-nucleation regime becomes shorter. All curves have been truncated at the point where phase separation occurs, signified by a distinct downwards bend. See Supplementary Figure 4 for detail at high pH and low Ca^{2+} concentrations.

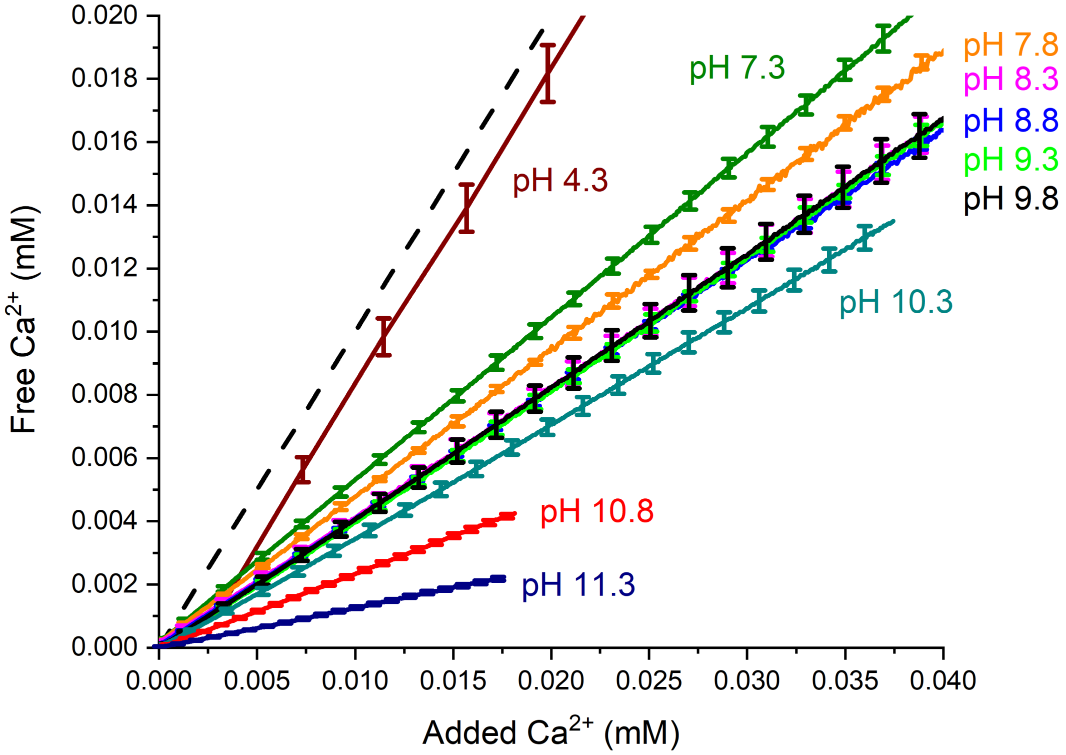

Supplementary Figure 4 Titration curves of calcium into phosphate buffer showing detail of low calcium concentrations. The curves measured at high pH can be distinguished, being shorter and lying below those of lower pH. The measured free calcium concentrations at pH 8.3 – 9.8 lie within the uncertainties of each other. Error bars are ± the standard deviation (N=3), only shown at selected data points for clarity.

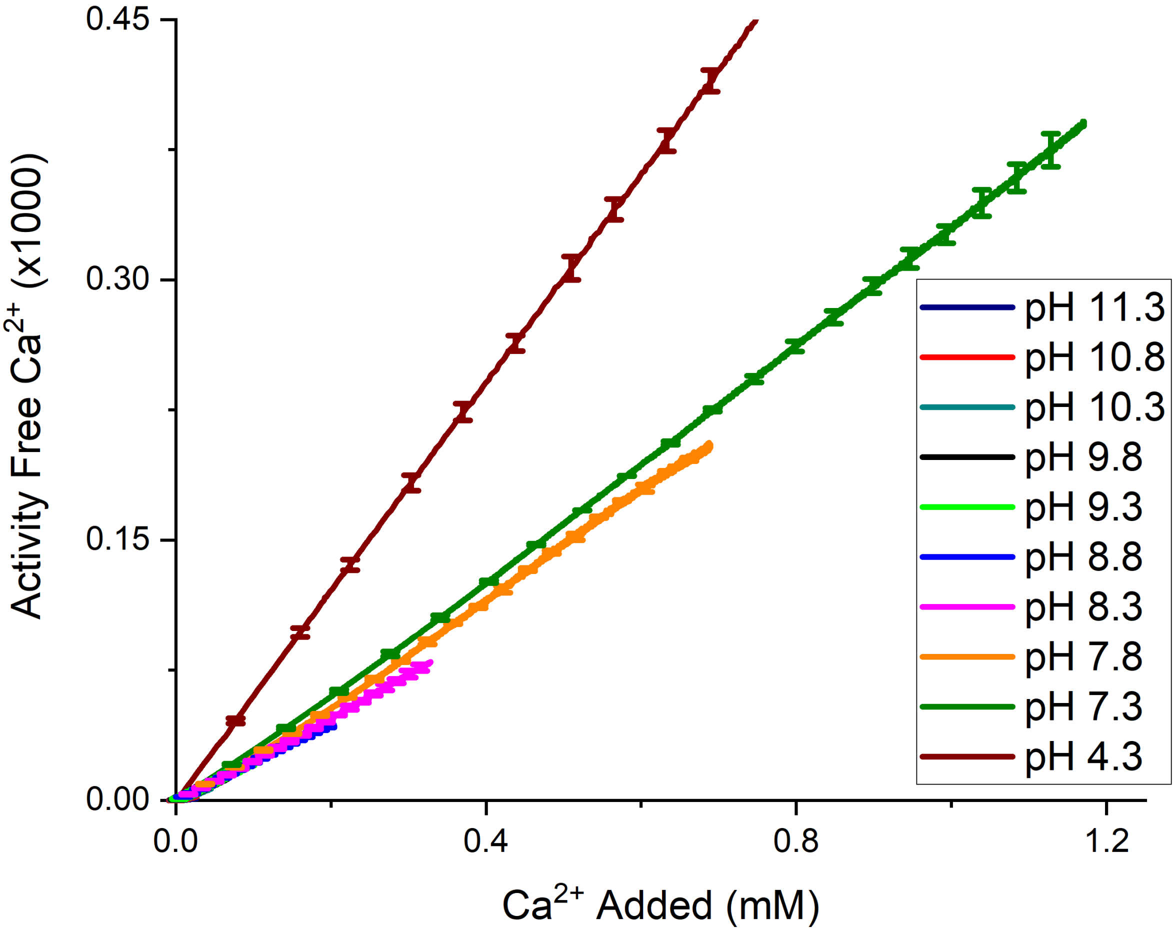

Supplementary Figure 5 Detected calcium converted to activity. Calculations were done using respective activity coefficients for each pH. Error bars represent standard deviation on three identical experiments, for each pH value 20 error bars are shown until the end of either the data or frame; curves extending above the y-axis limit may have less. Error bars are ± the standard deviation (N=3), only shown at selected data points for clarity.

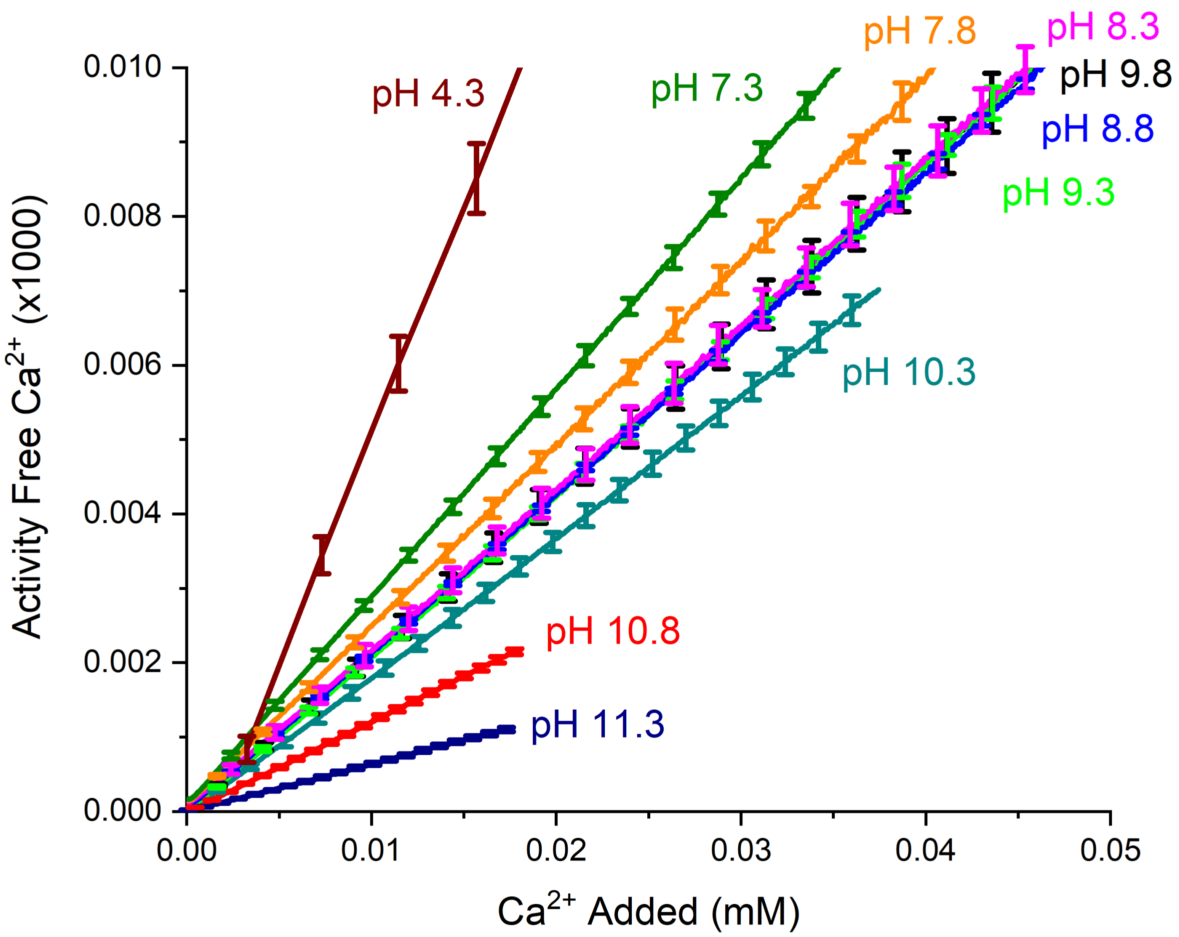

Supplementary Figure 6 Detail of titration free calcium converted to activity shown in Supplementary Figure 7. The pH dependence of calcium activity can be seen with greater amounts of calcium in solution at low pH. Error bars are ± the standard deviation (N=3), only shown at selected data points for clarity.

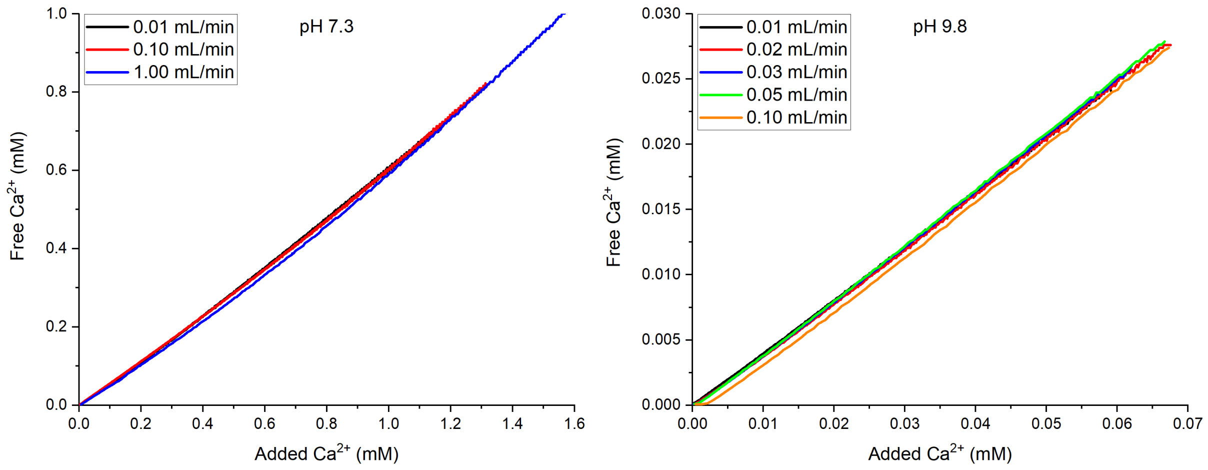

Supplementary Figure 7 Effect of titration addition rate on curves in the studied pre-nucleation regime. Left: Titration curves at pH 7.3. **Right:** Titration curves at pH 9.8. In all cases the measurements are nearly indistinguishable from each other; increasing the Ca^{2+} addition rate does not influence the ion association. The process of ion pairing is purely under thermodynamic control

in the experiments. The system is not influenced by kinetics and at equilibrium under the conditions studied (Supplementary Figure 8).

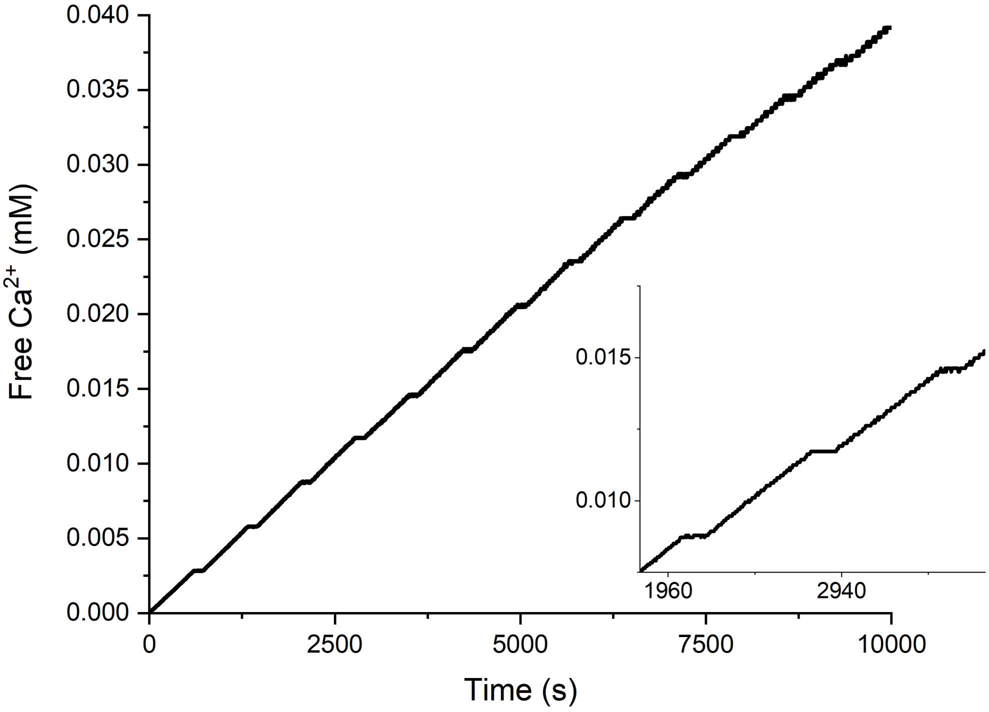

Supplementary Figure 8 Titration with paused addition. The pauses in the addition of calcium ions to the phosphate buffer can be seen as steps. During the pauses the concentration of free Ca^{2+} remains constant, while a linear increase is seen during the addition. This result shows that the system, and ion association, is at equilibrium during the titrations, the addition rate used (indeed any addition rate as shown in Supplementary Figure 7) does not affect the ion association. A decrease in the free calcium ion concentration would be seen during the pauses if the system needed to adjust itself due to addition being too fast – this is clearly not the case. The inset shows how consistent the plateaus are, with only measurement noise visible and no slope.

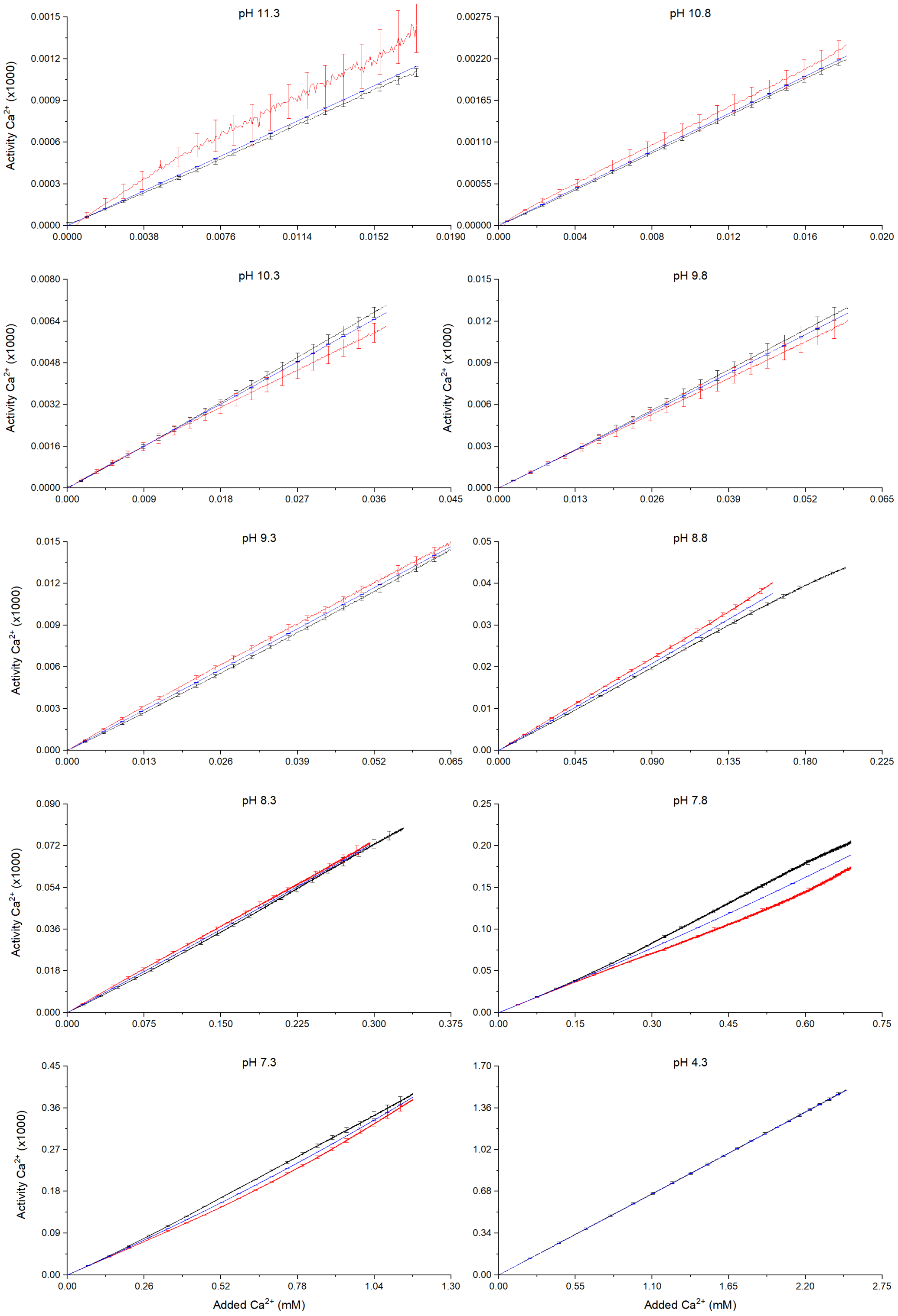

Supplementary Figure 9 Overview of the experimental calcium activities overlayed with calculated curves. The curves were calculated using the “Best Fit” constants with 4.5, 470, and 50,000 as K_1, K_2, and K_3, respectively. Curves (red) from the Direct Calculation are overlaid on the experimental titration data (black); the blue curves are from the Predictive Calculation. This set of ion association constants represents the best fit to the data. Only 20 total error bars are shown for each pH, representing the standard deviation of three identical experiments. The models also have error bars as the calculations were performed for each experiment individually and then averaged, this is continued for Supplementary Figures 10-12.

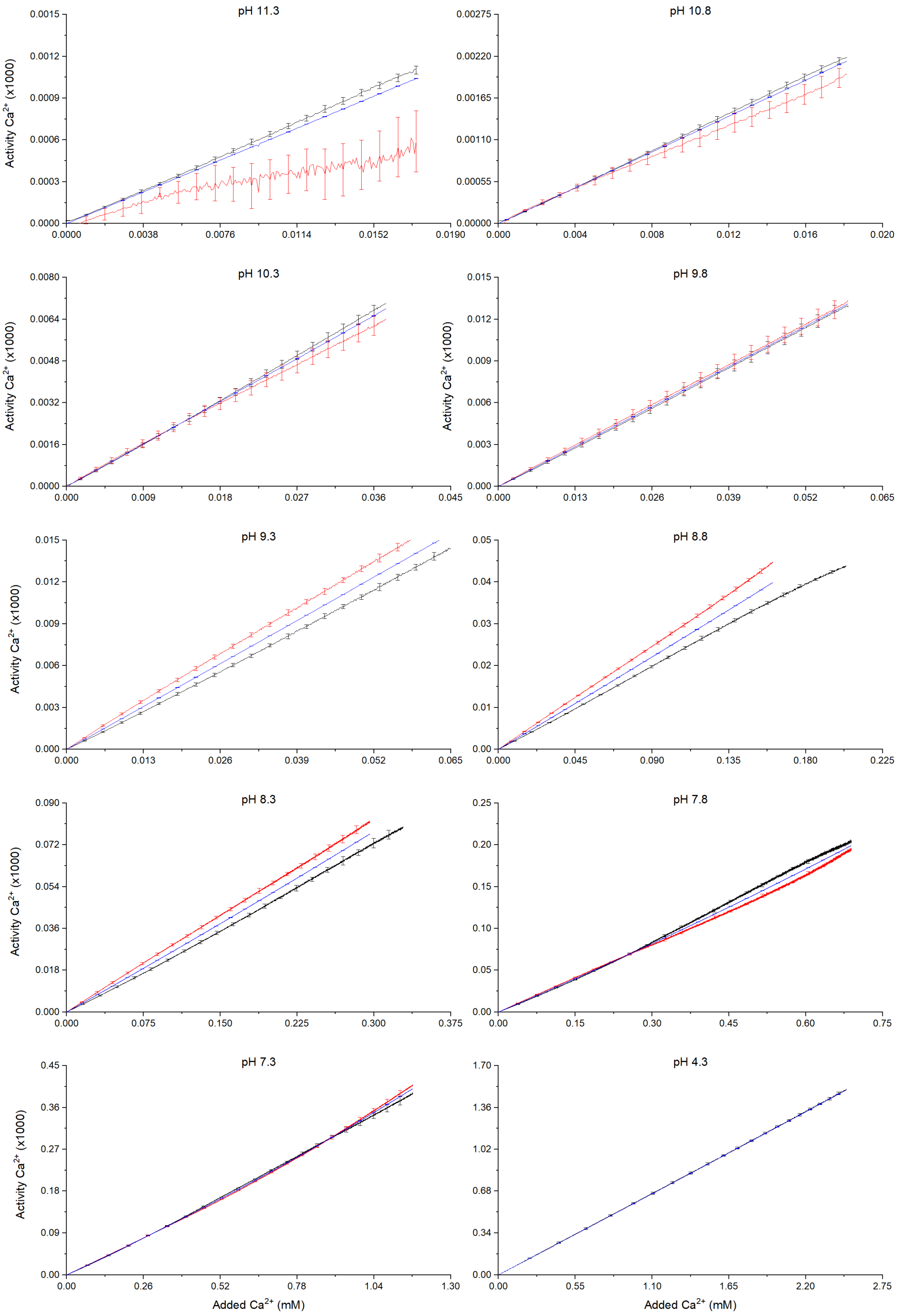

Supplementary Figure 10 Overview of the activity-based calculations at the limit of the error range from Table 1. The values 4.5, 420 and 58,000 are used for as K_1, K_2, and K_3, respectively ("Limit 1"); K_1 remains unchanged as the overall influence is small. Curves (red) from the Direct Calculation are overlaid on the experimental titration data (black) in the pre-nucleation regime for each pH value. Blue curves are the Predictive Calculation. This set of ion association constants represents the lowest K_2 and highest K_3 which describe the experimental data. Only 20 total error bars are shown for each pH, representing the standard deviation of three identical experiments.

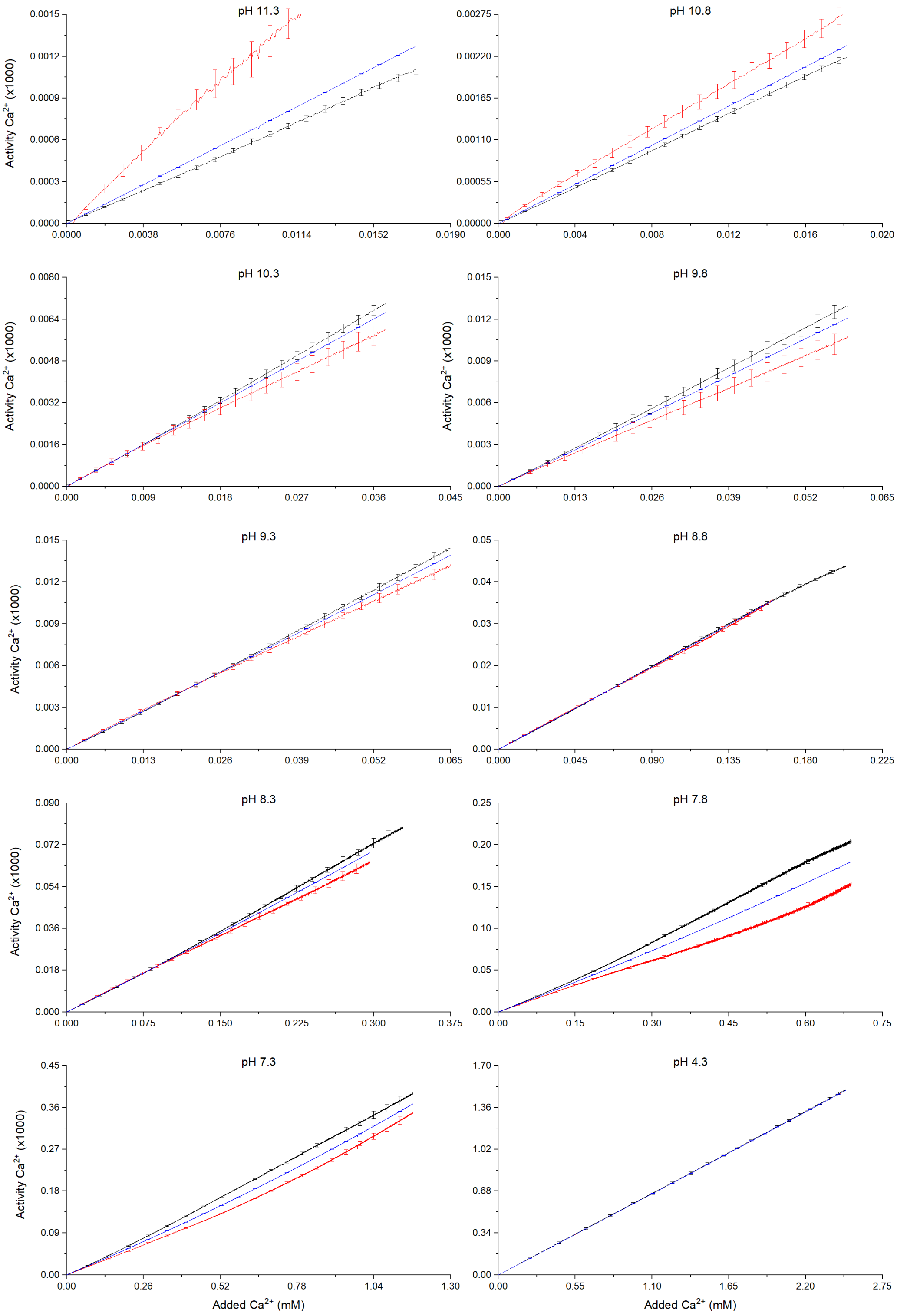

Supplementary Figure 11 Overview of the activity-based calculations at the limit of the error range from Table 1. The values 4.5, 520 and 42,000 are used for as K_1, K_2, and K_3, respectively ("Limit 2"); K_1 remains unchanged as the overall influence is small. Curves (red) from the Direct Calculation are overlaid on the experimental titration data (black) in the pre-nucleation regime for each pH value. Blue curves are the Predictive Calculation. This set of ion association constants represents the highest K_2 and lowest K_3 which describe the experimental data. Only 20 total error bars are shown for each pH, representing the standard deviation of three identical experiments.

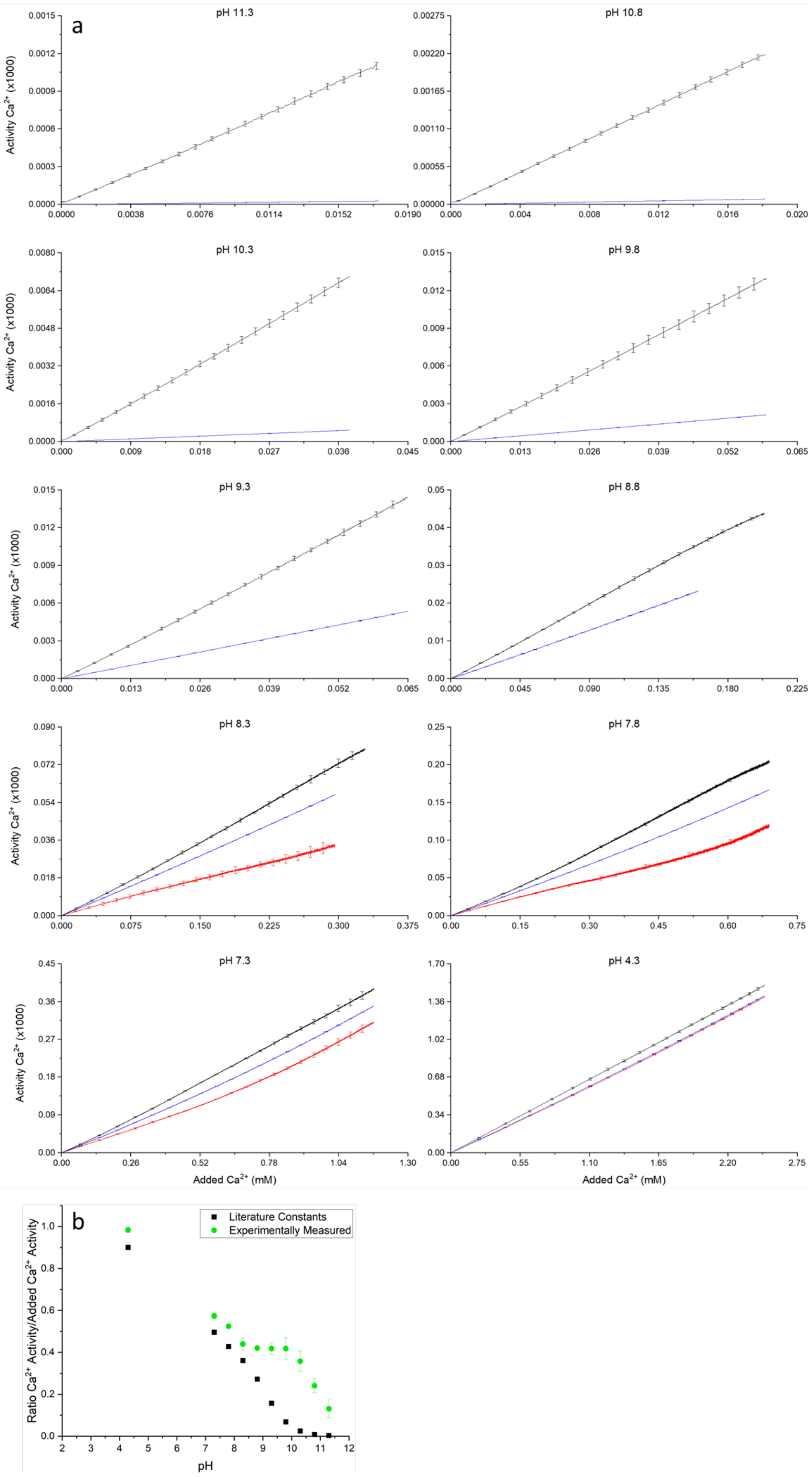
a
pH 11.3
pH 10.8
pH 10.3
pH 9.8
pH 9.3
pH 8.8
pH 8.3
pH 7.8
pH 7.3
pH 4.3
Activity Ca2+ (x1000)
Added Ca2+ (mM)
b
Literature Constants
Experimentally Measured
Ratio Ca2+ Activity/Added Ca2+ Activity
pH

Supplementary Figure 12 Overview of the activity-based calculations using the ion association constants from Chughtai *et al*.[1] a: Curves (red) from the Direct Calculation are overlayed on the experimental titration data (black) in the pre-nucleation regime for each pH value. Blue curves are the Predictive Calculation; lines are averages of three curves with twenty error bars showing the standard deviation. The literature binding constants overestimate the amount of ion pairs at all pH values- the calculated calcium activity is less than the experimentally determined activity in all cases. See Supplementary Section 2 for in-depth discussion of fitting and properties of the models. **b:** Comparison of the activity of free Ca^{2+} to the activity of added Ca^{2+} as calculated using the literature constants with the predictive calculation (black) to the experimentally determined values (green). The large influence of the large K_3 is seen starting around pH 9, where the prediction with literature values sees a decrease in the amount of free calcium ions compared to the measured values. The error bars on the experimental values is one standard deviation based on three titrations.

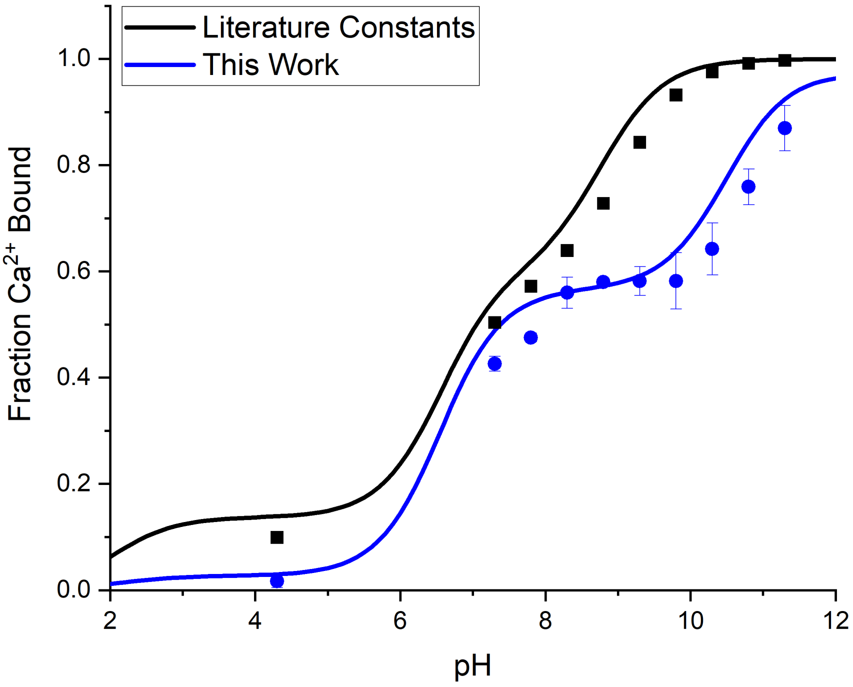

Supplementary Figure 13 Comparison of calcium bound as ion pairs. The amount of total calcium bound as ion pairs ($[CaHPO_4]^+$, $[CaHPO_4]^0$, and $[CaPO_4]^-$) in a solution 10 mM in phosphate and 0.2 mM in Ca^{2+} across the pH range 2-12 is shown, as calculated using the geochemical software package PHREEQC.[5, 6] The black line uses the calcium-phosphate ion pairing constants from Chughtai *et al*, 1968, while the blue line those determined in this work.[1] The smaller amount of bound calcium seen corresponds to the reduction of K_1 and K_3 by one and two orders of magnitude, respectively. Especially for K_3, the much later increase in bound calcium, after pH 10, compared to pH 8 using the previous values, shows how the revised ion association constants lead to a different solution composition. As pH increases, even the smaller K_3 predicts significant binding – this is due to the large fraction of PO_4^{3-} in solution under these conditions (see Supplementary Figure 14, phosphate speciation) The effect on the percent of bound calcium ions is naturally lower for K_1, as there is limited overall ion association at low pH values, although the reduced association can still be seen in the diagram. As the K_2 values are comparable, the percent of bound calcium predicted in the relevant pH range is similar. The fraction of bound calcium ions from the titrations and as determined by the predictive calculation using the literature ion association constants have been

overlayed as data points. The results agree well with the PHREEQC model, the slight discrepancy is likely due to the PHREEQC program taking ion association between all possible species into account; however, this difference is negligible to the conclusions. The error bars on the experimental values represent one standard deviation on the average of three titrations.

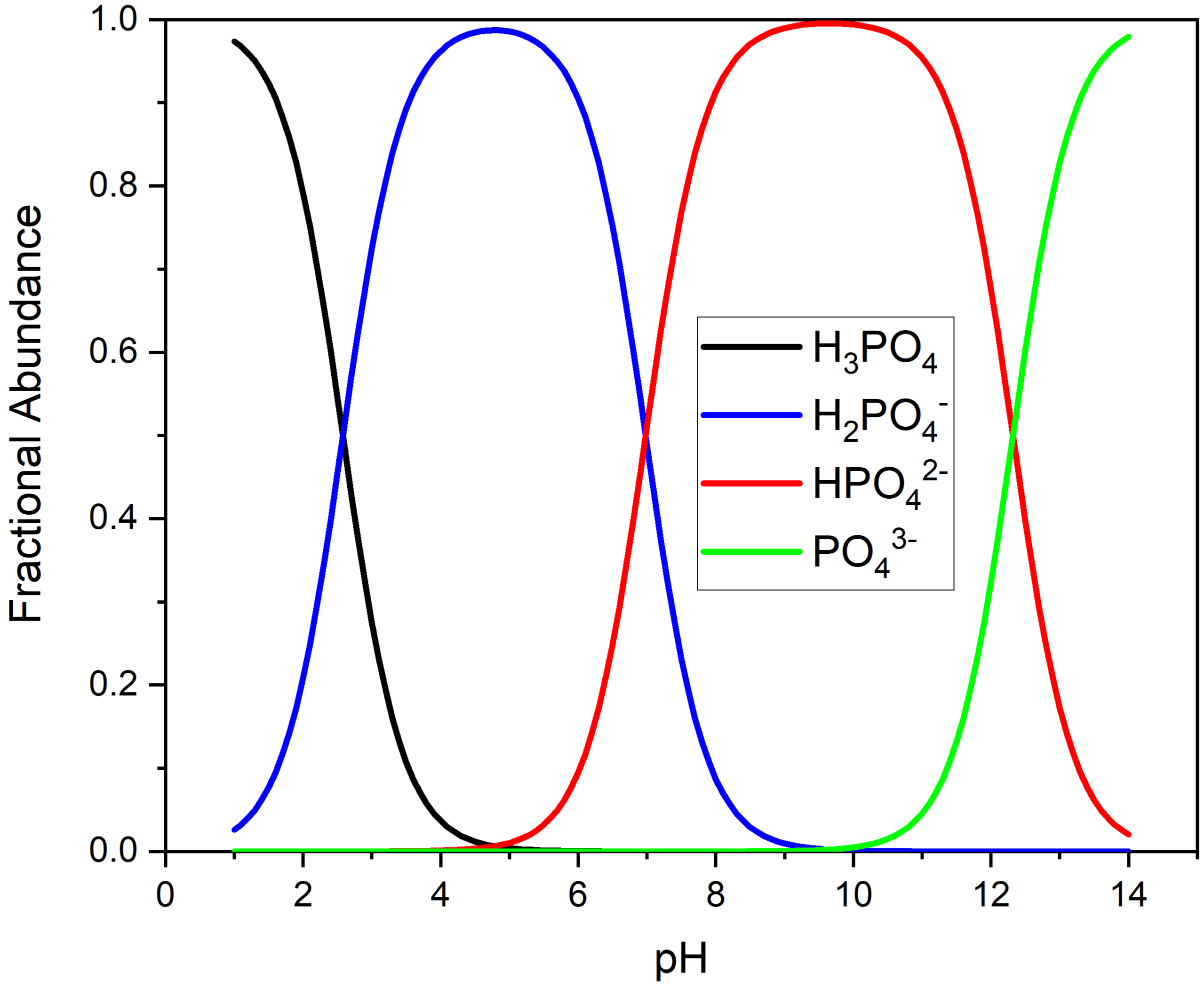

Supplementary Figure 14 pH dependent speciation of phosphate. The pKa values as determined in the titration have been used to calculate the fractional abundance of the ions. The amount of different phosphate species is useful in understanding the ion pair speciation, and the effects of the updated ion association constants, as seen in Figure 3 and Supplementary Figure 13.

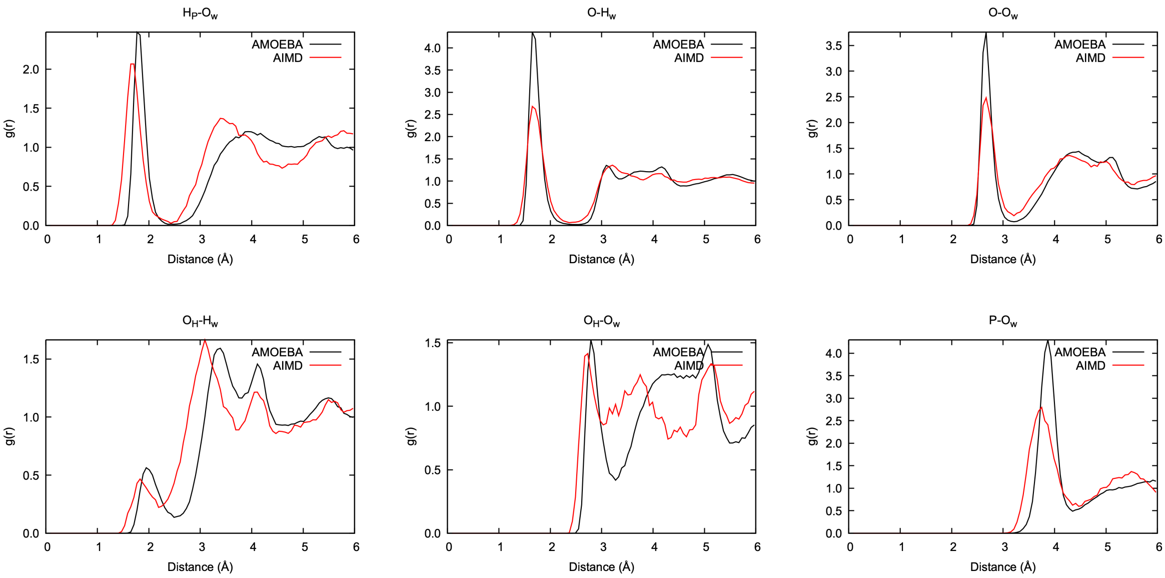

Supplementary Figure 15 Radial distribution function between HPO_4^{2-} and water. Distributions as computed using the AMOEBA force-field developed in this work and from *ab initio* molecular dynamics (AIMD).

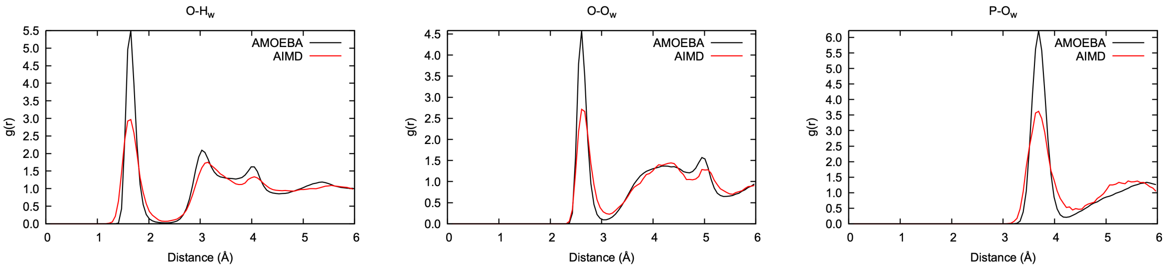

Supplementary Figure 16 Radial distribution function between PO_4^{3-} and water. Distributions as computed using the AMOEBA force field developed in this work and from *ab initio* molecular dynamics (AIMD).

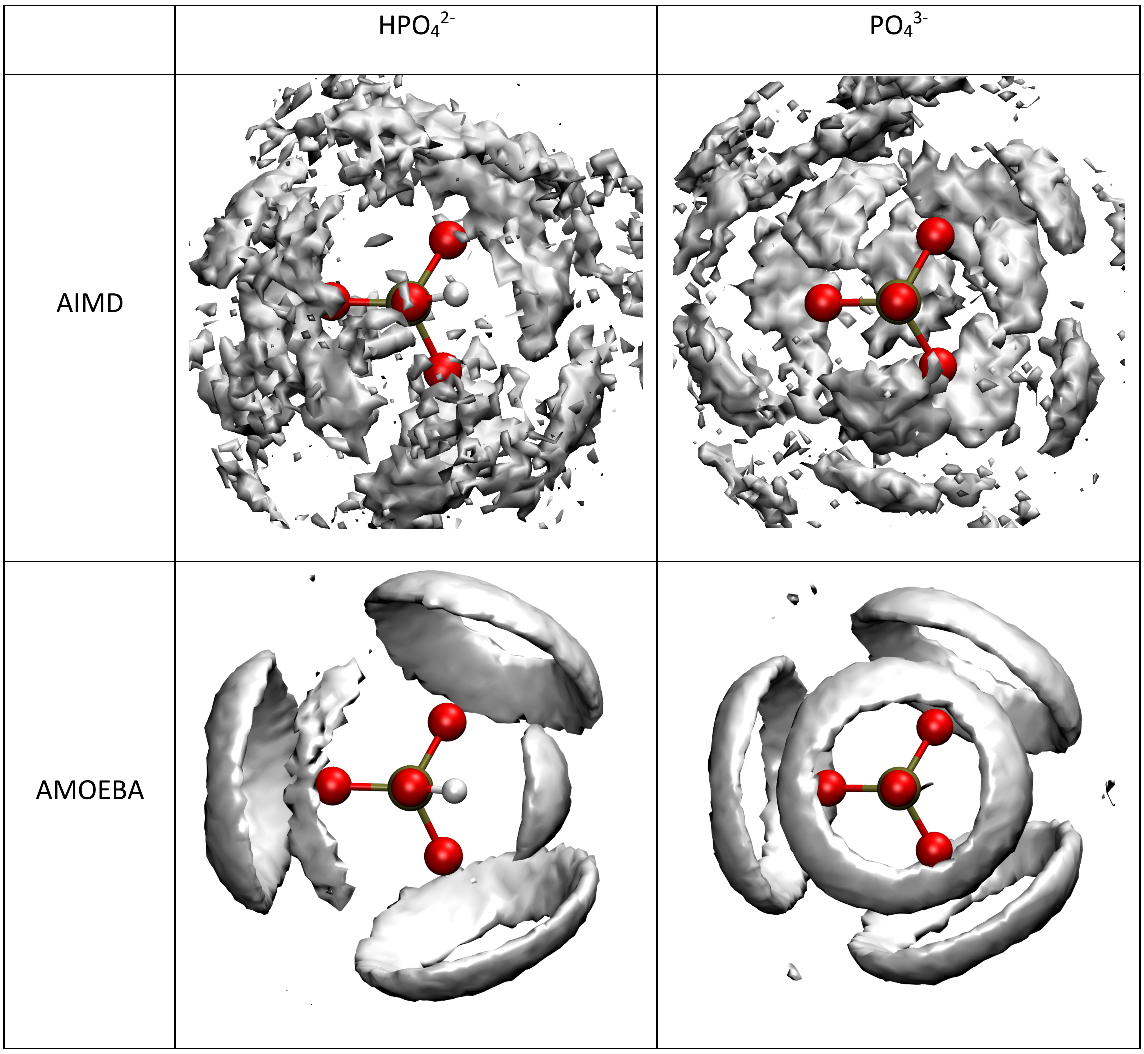

Supplementary Figure 17 Water density profile around the anions. Distributions were obtained from the AMOEBA and *ab initio* molecular dynamics (AIMD) simulations. The iso-surfaces were drawn at a density of 0.1 Å^{-3}.

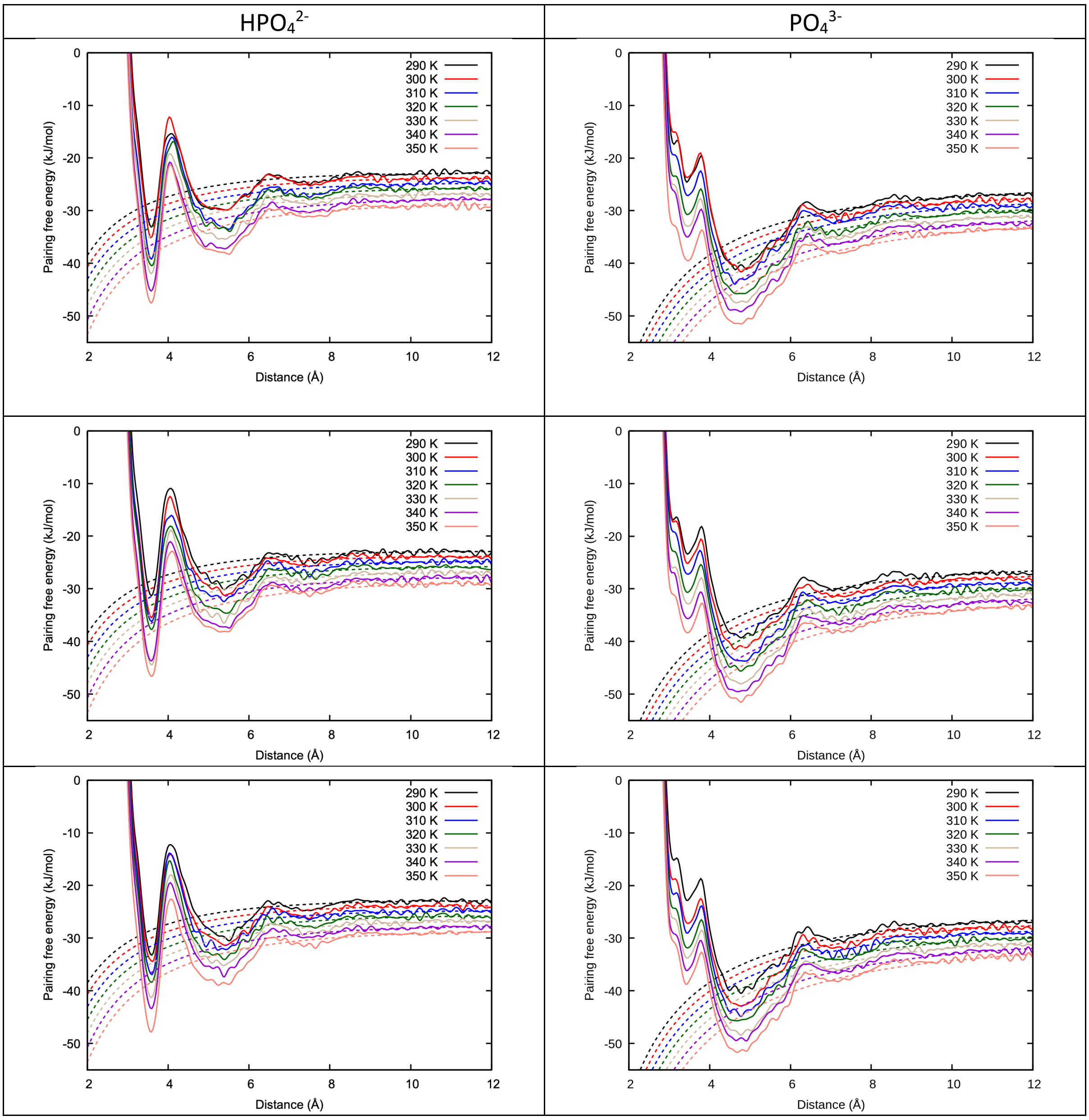

Supplementary Figure 18 Ion pairing free energies as a function of the Ca-P distance. The 1D free energy profiles were obtained from the AMOEBA simulations after integrating out the cation water coordination number.

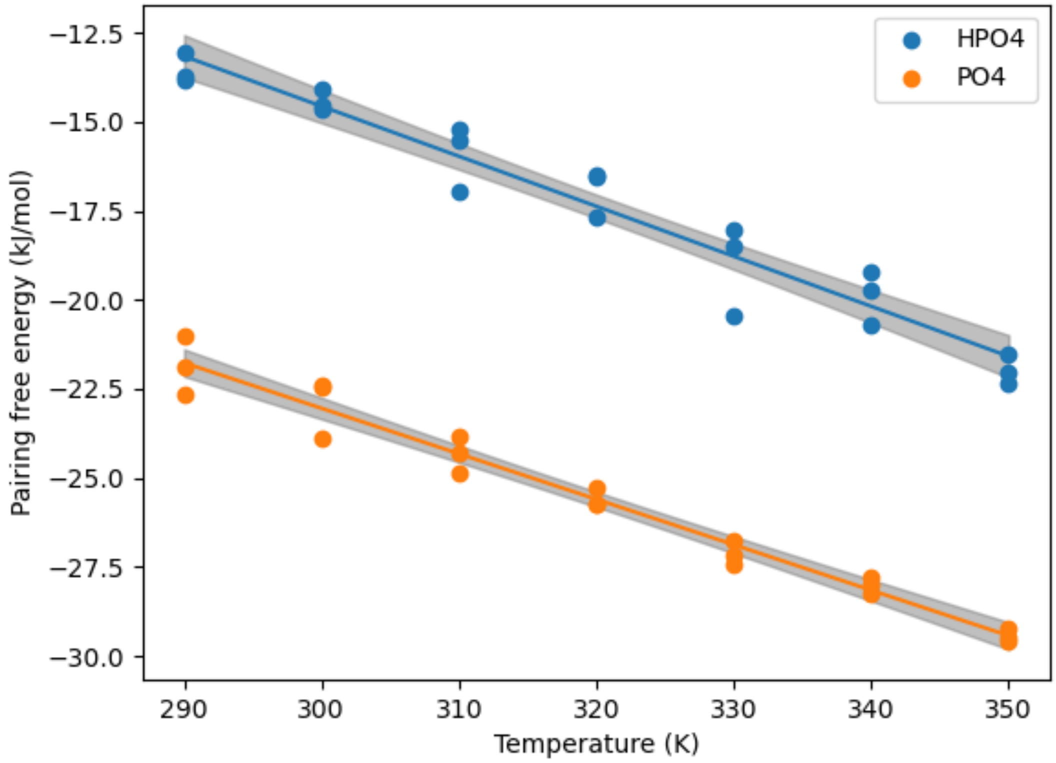

Supplementary Figure 19 Ion association free energy from AMOEBA-based simulations as a function of temperature. The grey region represents the 95% confidence interval.

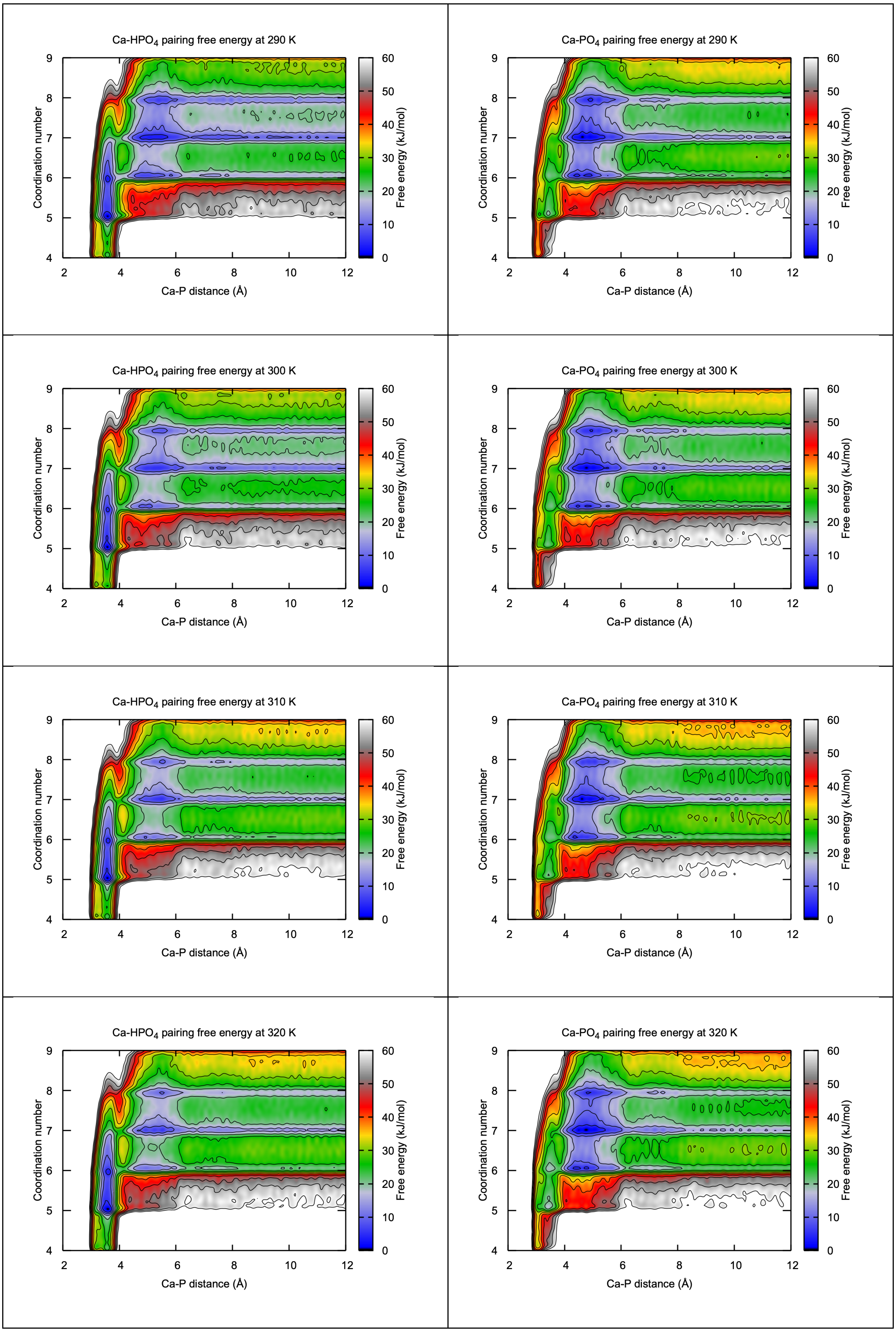
Ca-HPO$_4$ pairing free energy at 290 K
Ca-PO$_4$ pairing free energy at 290 K
Ca-HPO$_4$ pairing free energy at 300 K
Ca-PO$_4$ pairing free energy at 300 K
Ca-HPO$_4$ pairing free energy at 310 K
Ca-PO$_4$ pairing free energy at 310 K
Ca-HPO$_4$ pairing free energy at 320 K
Ca-PO$_4$ pairing free energy at 320 K
Coordination number
Ca-P distance (Å)
Free energy (kJ/mol)

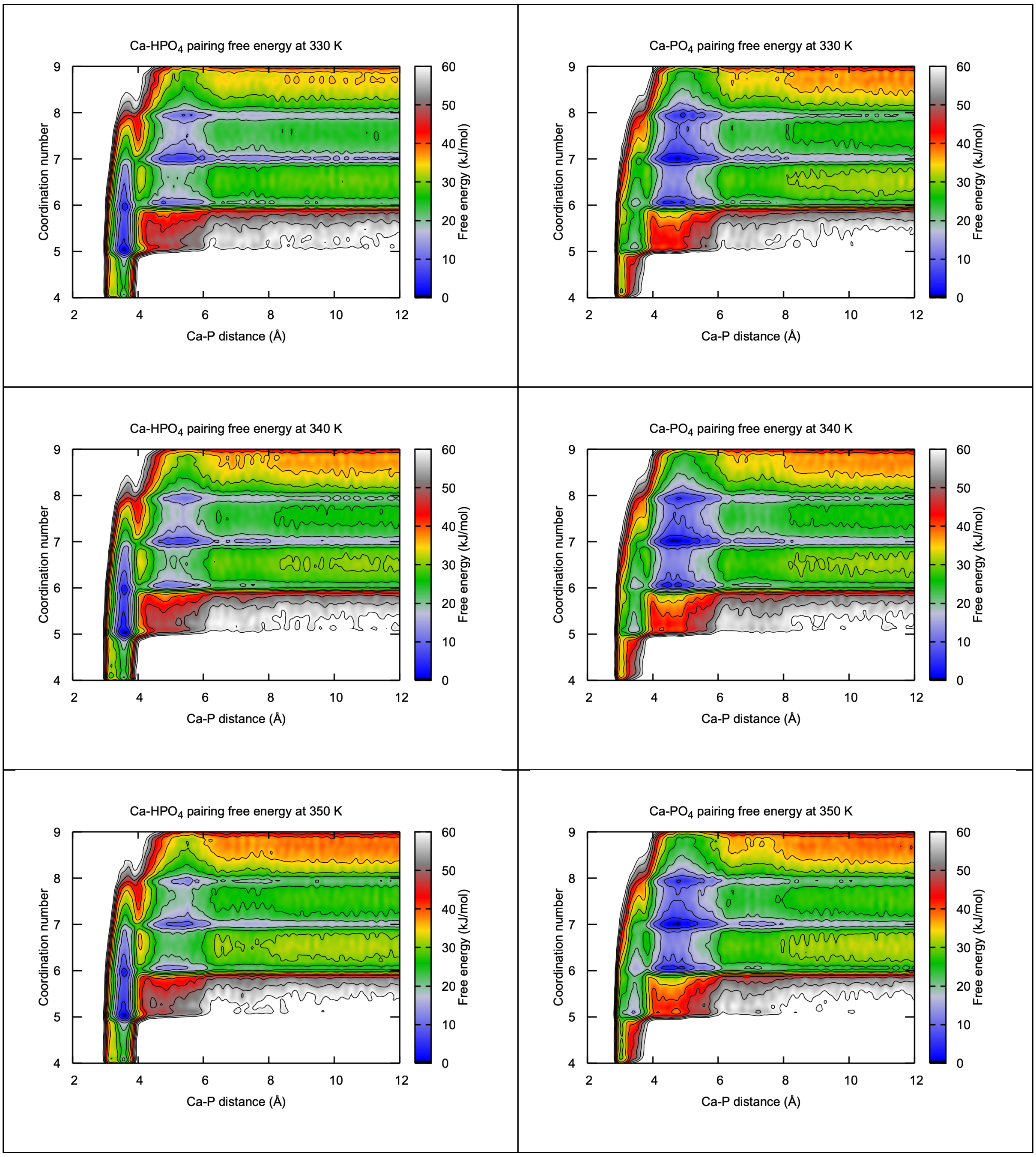

Supplementary Figure 20 Energy maps for ion association. Plots show 2D free energy map for the Ca-HPO_4^{2-} and Ca-PO_4^{3-} ion pair formation as a function of the Ca by water coordination number and the Ca-P distance computed using metadynamics and the AMOEBA polarisable force field developed in this work. The contour lines are drawn every $2k_BT$, and the free energy of the deepest minimum has been set to zero. The plots for only one of the three repeats are shown.

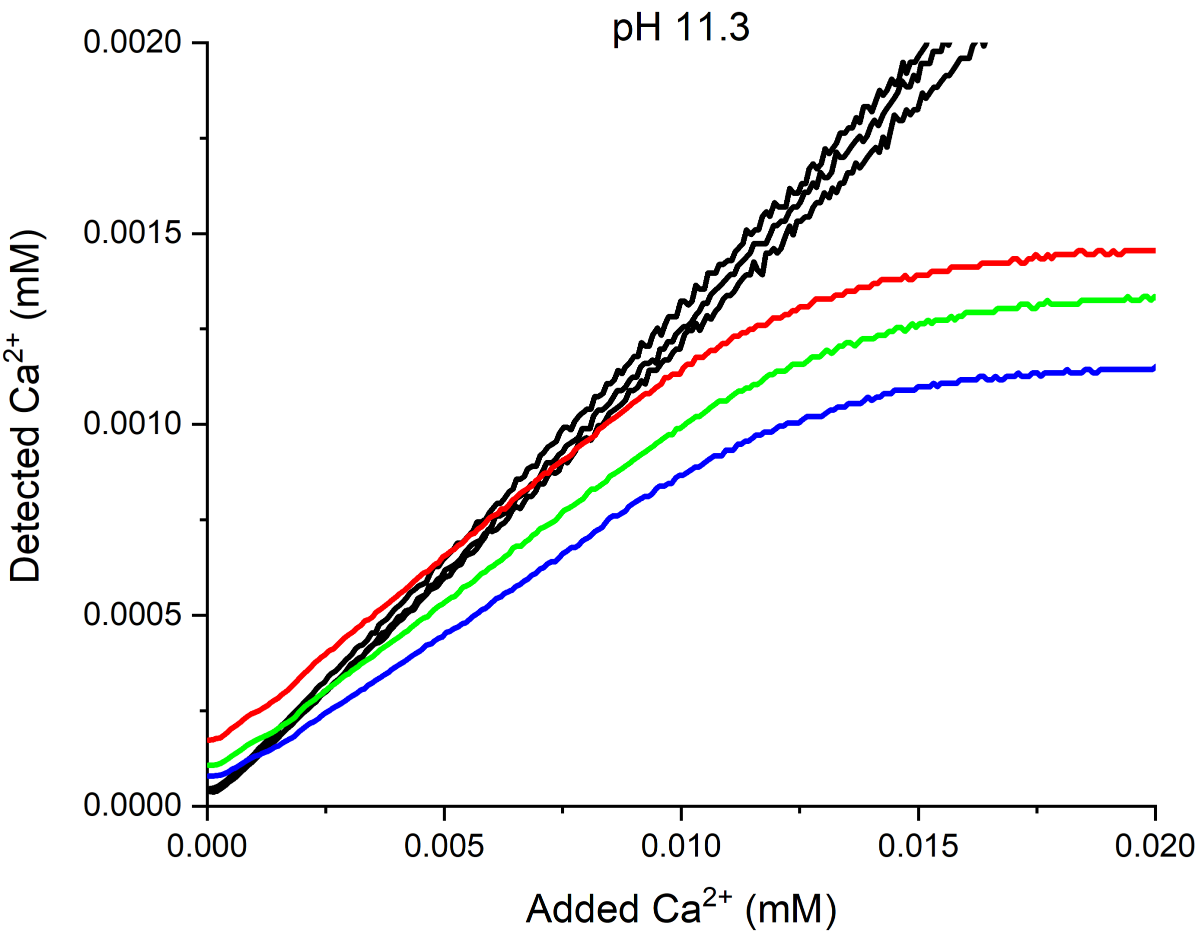

Supplementary Figure 21 Comparison of pH 11.3 titrations at 37°C and ambient conditions. Coloured titration curves at pH 11.3 and 37°C show the development of free calcium in solution as a function of added calcium for independent repetitions of single experiments. The black curves are the experiments at room temperature as shown in Supplementary Figures 3 and 4. Alongside the greater deviation between curves at elevated temperature, these measurements have a flatter slope, indicating greater binding. Titration into a plateau occurred even with acidification of the solutions. Additionally, the higher temperature leads to un-physical measurements at the start of the experiment, with readings starting at nearly 0.00025 mM Ca^{2+} in solution before any addition has occurred.

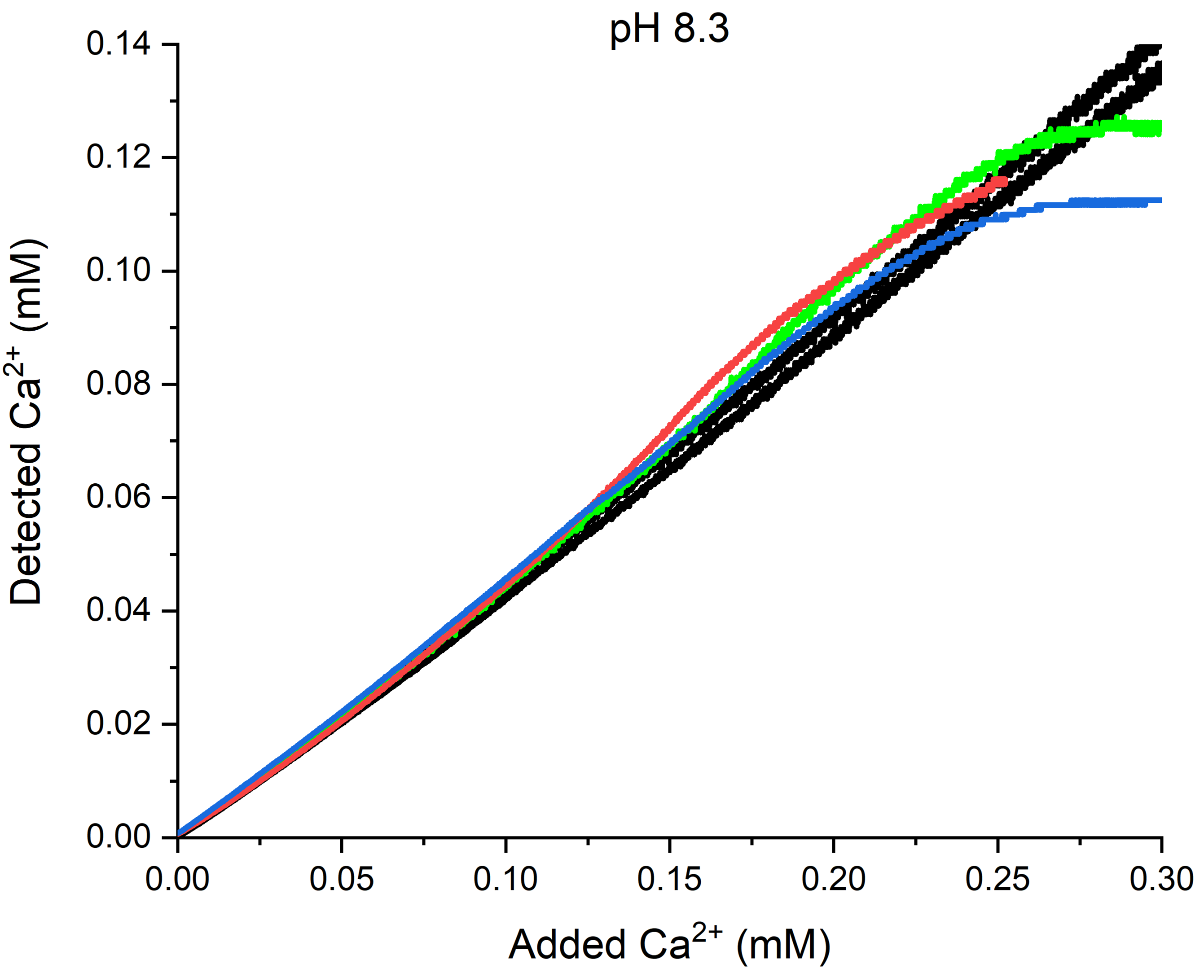

Supplementary Figure 22 Comparison of pH 8.3 titrations at 37°C and ambient conditions. Coloured curves show the amount of calcium in solution at pH 8.3 and 37°C. Black curves are the same experiment at room temperature (Supplementary Figures 3 and 4). The binding at both temperatures is comparable, however, the experiments at 37°C exhibit an earlier nucleation and a tendency to titrate into a plateau.

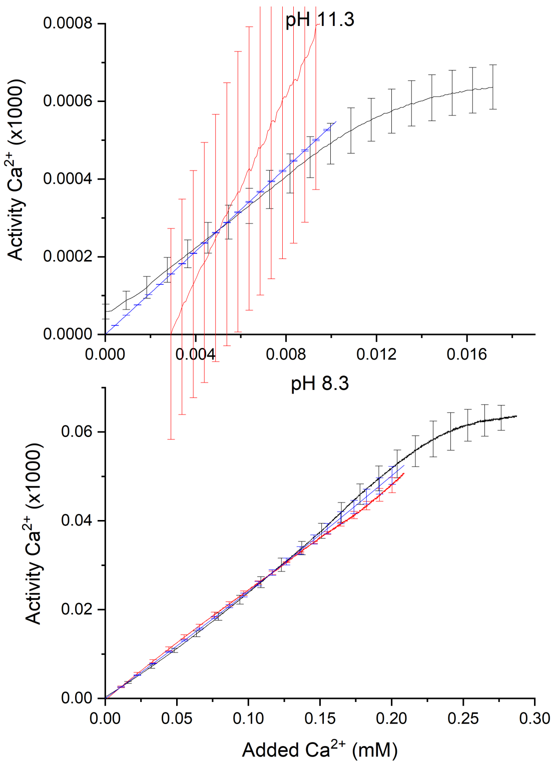

Supplementary Figure 23 Calculated free calcium activity at 37°C compared with experimental values (black). Ion association constants from Supplementary Table 3 are used. The red curve is the titration based Direct Calculation model while the blue is the Predictive calculation. Error bars represent standard deviation on three identical experiments, for clarity only 20 total error bars are shown for each pH. Increasing pH and temperature lead to an increase in experimental error; the Direct Calculation amplifies uncertainties as it relies on measured values leading to the large error bars at high pH (see discussion of fitting at ambient conditions, above).

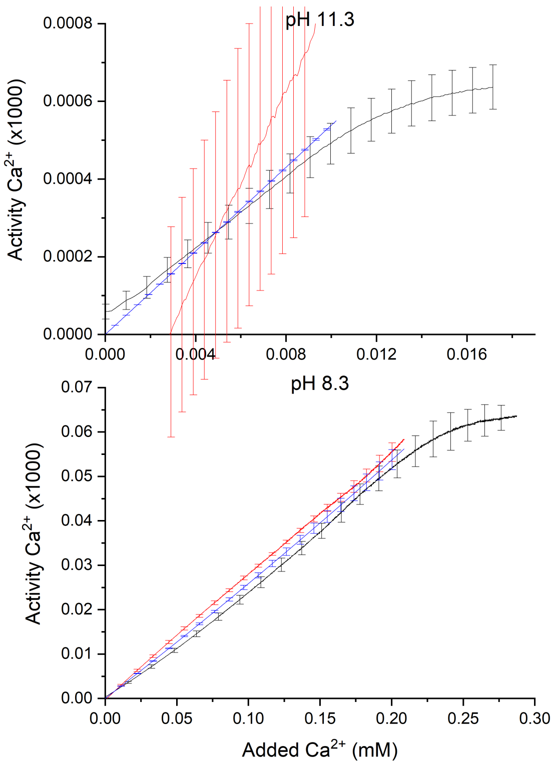

Supplementary Figure 24 Calculated free calcium activity at 37°C at the limit of the error range compared with experimental values (black). Ion association constants 4.5, 420 and 70,500 are used for K_1, K_2 and K_3, respectively. This group shows one extreme from the errors given in Supplementary Table 3. The red curve is the Direct Calculation while the blue is the Predictive Calculation. Error bars represent standard deviation on three identical experiments, for clarity only 20 total error bars are shown for each pH. Increasing pH and temperature lead to an increase in experimental error; the Direct Calculation amplifies uncertainties as it relies on measured values leading to the large error bars at high pH.

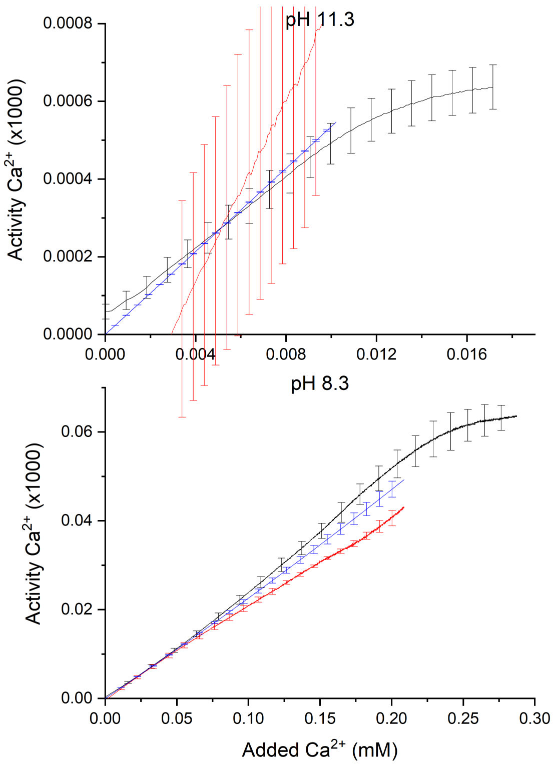

Supplementary Figure 25 Calculated free calcium activity at 37°C at the limit of the error range compared with experimental values (black). Ion association constants 4.5, 540 and 68,500 are used for K_1, K_2 and K_3, respectively. This group shows one extreme from the errors given in Supplementary Table 3. The red curve is the Direct Calculation while the blue is the Predictive Calculation. Error bars represent the standard deviation of three identical experiments with only 20 error bars shown per set for clarity. Increasing pH and temperature lead to an increase in experimental error; the Direct Calculation amplifies uncertainties as it relies on measured values leading to the large error bars at high pH.

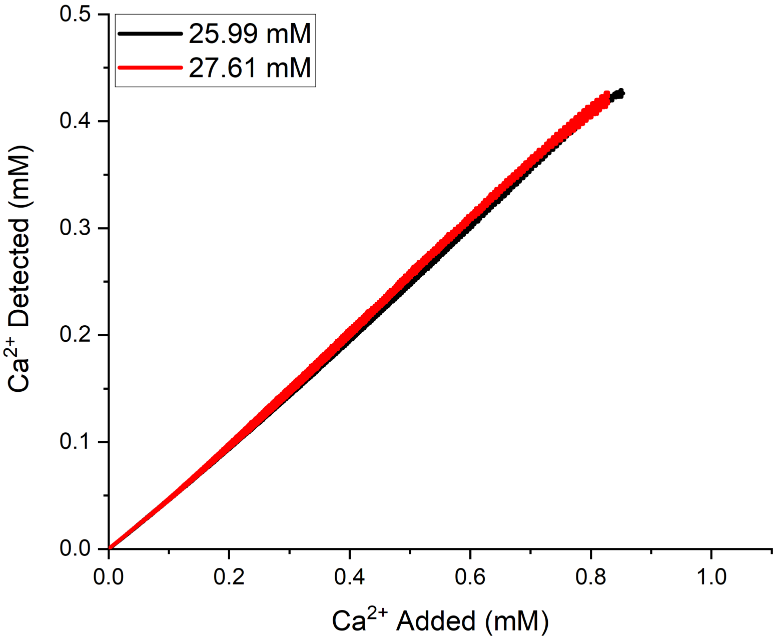

Supplementary Figure 26 Comparison between titrations performed at pH 7.8. Calibrations were performed either with a 25.99 mM (black) or 27.61 mM (red) NaCl solution. The curves lie nearly identically on top of each other, showing that deviations in calibration ionic strength do not significantly affect data quality.

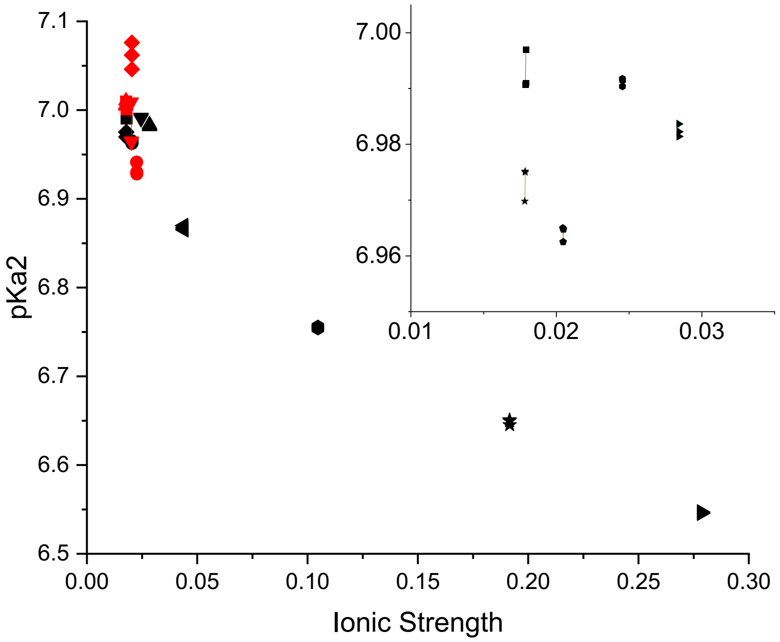

Supplementary Figure 27 pK_{a2} values and the corresponding ionic strength in the titration vessel. Black symbols indicate experiments performed using Method 1, red indicates all other methods. Inset shows results from Method 1 in the ionic strength range relevant in the calcium into phosphate titrations.

6. Supplementary Tables

Supplementary Table 1 Comparison of literature ion association values for the calcium phosphates. A spread in the values is evident; the paucity of reported values for K3 is also notable.

Author	Year	Temperature (C)	Comment	Method	K_1	Error	K_2	Error	K_3	Error
Greenwald[7]	1940	25		pH			155			
			I = 0.2 M, fixed with KCl				32	1.5		
Davies[8]	1953	25		Leaching	12	0.5	514	105		
Gosselin[9]	1953	37		Ion exchange/radiometry			72			
Smith[10]	1956	25	I = 0.2 M fixed with electrolyte	pH titration			50	0.7		
Greenwald[11]	1963						414	200		
Moreno[12]	1966	37.5		Leaching/Equilibrium	7.5	0.4	588	31		
Chughtai[1]	1968	25		pH	26.5	1.7	548	2.8	2.9E6	0.1E6
		37			31.9	1.6	681	2.8	3.46E6	0.2E6
Gregory[3]	1970	5	Chosen values using Davies equation for calc.	Leaching	9.69	1.83	479	57		
		15			5.23	1.29	283	36		
		25			3.67	0.98	364	36		
		37.5			3.28	1.48	431	74		
Childs[13]	1970	37	Various corr. Factors also available<br><br>McDowell *et al*, 1971 notes supersat. solutions	pH titration	4	2	20	1.6		
McDowell[4]	1971	5		Leaching and Equilibrations	5	1	240	30		
		15			10	1	190	20		
		25			10	1	380	40		
		37			11	1	390	90		
Atlas[14]	1976	20	I = 0.68 (seawater analogue)	pH titration	1.72		17.7		96100	
Zhang[15]	1991	37		pH titration	27.9	0.1	591	2	1.35E6	0.02E6
			Recalculating Chughtai *et al.*, 1968		24	0.9	630	3		
Zhang[16]	1992	37		Leaching	2.7	0.6				
Habraken[17]	2013		Average of reported values, I = 0.2 M	Titration			373	54		

Supplementary Table 2 Hydration free energies (kJ/mol) of HPO_4^{2-} and PO_4^{3-} at 300 K. The table shows values as predicted by the AMOEBA force field developed in this work compared with literature experimental and computational values.[18, 19, 20] Errors are in the accompanying data file.

	AMOEBA	Marcus[19, 20]	Florián&Warshel[18]
Ca^{2+}	−1495	−1505/−1527	−1581
HPO_4^{2-}	−1384	− /−1366	−1025
PO_4^{3-}	−2762	−2765/−2753	−2242

Supplementary Table 3 Overview of the ion association constants for the calcium phosphate system at 37°C. The ion association constants and the corresponding errors were determined identically as for the titrations at room temperature (see Methods). The calculated activities with these constants, and those at the limits of the error range are overlaid onto the experimental data in Supplementary Figures 23 to 25.

Ion Association 37°C	1	2	3
K_i	4.5	480	69,500
Error	±2.4	±60	±1000

Supplementary Table 4 Summary of experimental conditions. The table gives an overview of the concentrations, acidities and NaCl amounts used to adjust the ionic strength for the added calcium solutions and sodium hydroxide used to maintain a constant pH in both acidified experiments and the corresponding calcium calibrations.

	Experiment		Calibration		
pH	Ca (M)	NaOH (M)	Cal. NaCl (M)	Cal. Ca (M)	Cal. NaOH (M)
11.3	0.005 + 0.05 HCl	0.1	0.03361	0.005 + 0.05 HCl	0.1
10.8	0.005 + 0.05 HCl	0.1	0.03119	0.005 + 0.05 HCl	0.1
10.3	0.005 + 0.05 HCl	0.1	0.03037	0.005 + 0.05 HCl	0.1
9.8	0.005 + 0.05 HCl	0.1	0.03006	0.005 + 0.05 HCl	0.1
9.3	0.005 + 0.01 HCl	0.1	0.03000	0.005 + 0.01 HCl	0.1
8.8	0.005 + 0.001 HCl	0.1	0.02987	0.005 + 0.0005 NaCl	0.01 + 0.045 NaCl
8.3	0.005 + 0.001 HCl	0.1	0.02945	0.005 + 0.0005 NaCl	0.01 + 0.045 NaCl
7.8	0.005 + 0.01 HCl	0.1	0.02830	0.005 + 0.005 NaCl	0.01 + 0.045 NaCl
7.3	0.005	0.1	0.02467	0.005	0.01
4.3	0.005	0.1	0.01502	0.005	0.1

Supplementary Table 5 Summary of pK_a titrations. The table gives an overview of multiple methods used to determine pK_a values of phosphoric acid in the titration setup. Optimal and slow rates are pre-set options in the software, methods 5 and 6 were performed with dilution control.

Method	PO4 Soln. (g)	NaOH (M)	Rate
1	25	0.1	Optimal
2	25	0.1	Slow
3	25	1	Optimal
4	50	0.1	Optimal
5	25	1	0.01 mL/min
6	25	0.1	0.01 mL/min

7. Supplementary References

1. Chughtai AR, Marshall R, Nancollas GH. Complexes in calcium phosphate solutions. *J. Phys. Chem.* **72**, 208-211 (1968).

2. Smith RM, Martell AE. *Critical stability constants: inorganic complexes*. Springer (1976).

3. Gregory TM, Moreno EC, Brown WE. Solubility of $CaHPO_4 \cdot 2H_2O$ in the system $Ca(OH)_2$-H_3PO_4-H_2O at 5, 15, 25, and 37.5° C. *J. Res. Natl. Bur. Stand.* **74**, 461-475 (1970).

4. McDowell H, Brown WE, Sutter J. Solubility study of calcium hydrogen phosphate. Ion-pair formation. *Inorg. Chem.* **10**, 1638-1643 (1971).

5. Parkhurst DL, Appelo C. Description of input and examples for PHREEQC version 3—a computer program for speciation, batch-reaction, one-dimensional transport, and inverse geochemical calculations. *US geological survey techniques and methods* **6**, 497 (2013).

6. Parkhurst DL, Thorstenson DC, Plummer LN. *PHREEQE: A computer program for geochemical calculations*. US Geological Survey, Water Resources Division (1982).

7. Greenwald I, Redish J, Kibrick AC. The dissociation of calcium and magnesium phosphates. *J. Biol. Chem.* **135**, 65-76 (1940).

8. Davies C, Hoyle B. The Interaction of Calcium Ions with Some Phosphate and Citrate Buffers. *J. Chem. Soc.*, 4134-4136 (1953).

9. Gosselin RE, Coghlan E. The stability of complexes between calcium and orthophosphate, polymeric phosphate, and phytate. *Arch. Biochem. Biophys.* **45**, 301-311 (1953).

10. Smith RM, Alberty RA. The apparent stability constants of Ionic complexes of various adenosine phosphates with divalent cations1, 2. *J. Am. Chem. Soc.* **78**, 2376-2380 (1956).

11. Greenwald I. The instability constant and hydrogen ion dissociation of calcium hydrogen phosphate. *J. Phys. Chem.* **67**, 2853-2854 (1963).

12. Moreno EC, Gregory T, Brown WE. Solubility of $CaHPO_4 \cdot 2H_2O$ and Formation of Ion Pairs in System $Ca(OH)_2$-H_3PO_4-H_2O at 37.5 Degrees C. *J. Res. Nat. Bur. Stand., Sect. A* **70**, 545-+ (1966).

13. Childs C. Potentiometric study of equilibriums in aqueous divalent metal orthophosphate solutions. *Inorg. Chem.* **9**, 2465-2469 (1970).

14. Atlas E, Culberson C, Pytkowicz R. Phosphate association with Na+, Ca2+ and Mg2+ in seawater. *Mar. Chem.* **4**, 243-254 (1976).

15. Zhang J, Ebrahimpour A, Nancollas G. Ion association in calcium phosphate solutions at 37 C. *J. Solution Chem.* **20**, 455-465 (1991).

16. Zhang J, Nancollas GH. Interpretation of dissolution kinetics of dicalcium phosphate dihydrate. *J. Cryst. Growth* **125**, 251-269 (1992).

17. Habraken WJEM*, et al.* Ion-association complexes unite classical and non-classical theories for the biomimetic nucleation of calcium phosphate. *Nat. Commun.* **4**, 1507 (2013).

18. Florián J, Warshel A. Calculations of hydration entropies of hydrophobic, polar, and ionic solutes in the framework of the Langevin dipoles solvation model. *J. Phys. Chem. B* **103**, 10282-10288 (1999).

19. Marcus Y. *Ions in Solution and their Solvation*. John Wiley & Sons (2015).

20. Marcus Y. Thermodynamics of solvation of ions. Part 5.—Gibbs free energy of hydration at 298.15 K. *J. Chem. Soc., Faraday Trans.* **87**, 2995-2999 (1991).
