# Supplementary material for: Redefined ion association constants have consequences for calcium phosphate nucleation and biomineralization
Source: Nat Commun. 2024 Apr 18;15:3359. doi: 10.1038/s41467-024-47721-7 (PMC11026415; doi:10.1038/s41467-024-47721-7)
Supplement: Supplementary file 2 — Description of Additional Supplementary Files [file 41467_2024_47721_MOESM2_ESM.pdf]

Supplementary Movie 1:
The curves were calculated using the displayed constants as K_1, K_2, and K_3, respectively. Curves (red) from the Direct Calculation are overlaid on the experimental titration data (black); the blue curves are from the Predictive Calculation. This set of ion association constants represents the best fit to the data. Only 20 total error bars are shown for each pH, representing the standard deviation of three identical experiments. The models also have error bars as the calculations were performed for each experiment individually and then averaged. Supplementary Movie 1 changes K_3 while keeping K_2 constant. The effect of the varied constant can be seen.

Supplementary Movie 2:
The curves were calculated using the displayed constants as K_1, K_2, and K_3, respectively. Curves (red) from the Direct Calculation are overlaid on the experimental titration data (black); the blue curves are from the Predictive Calculation. This set of ion association constants represents the best fit to the data. Only 20 total error bars are shown for each pH, representing the standard deviation of three identical experiments. The models also have error bars as the calculations were performed for each experiment individually and then averaged. Supplementary Movie 2 changes K_2 while keeping K_3 constant. The effect of the varied constant can be seen.
